# A novel function of STAT3β in suppressing interferon response improves outcome in acute myeloid leukemia

**DOI:** 10.1038/s41419-024-06749-9

**Published:** 2024-05-28

**Authors:** Sophie Edtmayer, Agnieszka Witalisz-Siepracka, Bernhard Zdársky, Kerstin Heindl, Stefanie Weiss, Thomas Eder, Sayantanee Dutta, Uwe Graichen, Sascha Klee, Omar Sharif, Rotraud Wieser, Balázs Győrffy, Valeria Poli, Emilio Casanova, Heinz Sill, Florian Grebien, Dagmar Stoiber

**Affiliations:** 1 Division Pharmacology, Department of Pharmacology, Physiology and Microbiology, Karl Landsteiner University of Health Sciences, Krems, Austria; 2 Institute for Medical Biochemistry, University of Veterinary Medicine Vienna, Vienna, Austria; 3 Division of Oncology, Medical University of Graz, Graz, Austria; 4 Division Biostatistics and Data Science, Department of General Health Studies, Karl Landsteiner University of Health Sciences, Krems, Austria; 5 Institute for Vascular Biology, Centre for Physiology and Pharmacology, Medical University of Vienna, Vienna, Austria; 6 Christian Doppler Laboratory for Immunometabolism and Systems Biology of Obesity-Related Diseases (InSpiReD), Vienna, Austria; 7 Division of Oncology, Department of Medicine I, Medical University of Vienna, Vienna, Austria; 8 Ludwig Boltzmann Institute for Hematology and Oncology, Medical University of Vienna, Vienna, Austria; 9 Department of Bioinformatics, Semmelweis University, Budapest, Hungary; 10 Department of Biophysics, Medical School, University of Pecs, Pecs, Hungary; 11 Cancer Biomarker Research Group, Institute of Molecular Life Sciences, HUN-REN Research Centre for Natural Sciences, Budapest, Hungary; 12 Department of Molecular Biotechnology and Health Sciences, University of Turin, Turin, Italy; 13 Department of Pharmacology, Center of Physiology and Pharmacology & Comprehensive Cancer Center (CCC), Medical University of Vienna, Vienna, Austria; 14 Division of Hematology, Medical University of Graz, Graz, Austria; 15 St. Anna Children’s Cancer Research Institute (CCRI), Vienna, Austria; 16 CeMM Research Center for Molecular Medicine of the Austrian Academy of Sciences, Vienna, Austria

**Keywords:** Acute myeloid leukaemia, Tumour-suppressor proteins

## Abstract

Signal transducer and activator of transcription 3 (STAT3) is frequently overexpressed in patients with acute myeloid leukemia (AML). STAT3 exists in two distinct alternatively spliced isoforms, the full-length isoform STAT3α and the C-terminally truncated isoform STAT3β. While STAT3α is predominantly described as an oncogenic driver, STAT3β has been suggested to act as a tumor suppressor. To elucidate the role of STAT3β in AML, we established a mouse model of STAT3β-deficient, *MLL-AF9*-driven AML. STAT3β deficiency significantly shortened survival of leukemic mice confirming its role as a tumor suppressor. Furthermore, RNA sequencing revealed enhanced STAT1 expression and interferon (IFN) signaling upon loss of STAT3β. Accordingly, STAT3β-deficient leukemia cells displayed enhanced sensitivity to blockade of IFN signaling through both an IFNAR1 blocking antibody and the JAK1/2 inhibitor Ruxolitinib. Analysis of human AML patient samples confirmed that elevated expression of IFN-inducible genes correlated with poor overall survival and low *STAT3β* expression. Together, our data corroborate the tumor suppressive role of STAT3β in a mouse model in vivo. Moreover, they provide evidence that its tumor suppressive function is linked to repression of the STAT1-mediated IFN response. These findings suggest that the *STAT3β/α* mRNA ratio is a significant prognostic marker in AML and holds crucial information for targeted treatment approaches. Patients displaying a low *STAT3β/α* mRNA ratio and unfavorable prognosis could benefit from therapeutic interventions directed at STAT1/IFN signaling.

## Introduction

Acute myeloid leukemia (AML) is an aggressive form of leukemia occurring both in children and adults. The development of effective therapeutic strategies is complex due to the wide array of cytogenetic and molecular abnormalities observed in patients. Although targeted therapies with small molecule inhibitors of FLT3, BCL2, and IDH1/2 have improved disease outcomes for several AML subtypes, the 5-year survival rate remains only around 30% [1]. Thus, novel treatment combinations and prognostic markers are urgently needed.

Signal transducer and activator of transcription 3 (STAT3) is an important transcription factor regulating stem cell self-renewal, hematopoiesis, and inflammation [2]. Aberrant STAT3 signaling promotes proliferation and survival in various malignancies and therefore has been explored as a therapeutic target [3]. Moreover, enhanced STAT3 activity is associated with poor prognosis and increased resistance to chemotherapy [4–8]. In AML, constitutive STAT3 activation was reported in AML patient samples and cell lines and contributes to leukemia cell survival, uncontrolled proliferation, and evasion of apoptosis [4, 6–9]. Importantly, alternative splicing gives rise to two isoforms with distinct functions contributing to the diverse roles of STAT3 in cancer [10–12]. While STAT3α represents the full-length isoform, STAT3β is truncated due to an alternative acceptor site causing a C-terminal deletion of 55 amino acids. Consequently, STAT3β lacks the transactivation domain including the phosphorylation site serine 727 [13]. Therefore, STAT3β was initially considered as a negative regulator of STAT3α [14]. However, studies demonstrated that STAT3β plays a relevant role as a transcriptional regulator as it was capable of rescuing the embryonic lethality in STAT3α-deficient mice [11, 12]. Furthermore, STAT3β was reported to induce transcription of a distinct set of genes in a STAT3α-independent manner [11, 12, 15, 16]. In more recent studies, STAT3β gained attention as a tumor suppressive molecule in melanoma [17], lung cancer [18], and esophageal squamous cell carcinoma [16]. In our previous study, we demonstrated that a higher *STAT3β/α* mRNA ratio correlates with prolonged overall survival (OS) in AML patients [19]. Moreover, overexpression of STAT3β in murine leukemia cells delayed disease progression in mice [19]. However, the molecular mechanisms behind its protective role remained elusive.

Here, we provide insights into the cellular processes resulting in the tumor suppressive function of STAT3β in AML and assign a link between STAT3 isoform expression and interferon (IFN) signaling in leukemia cells. Absence of STAT3β led to an enhanced IFN response and unfavorable disease outcomes, both in an AML mouse model and in primary AML patient samples. Furthermore, STAT3β-deficient leukemia cells were specifically vulnerable to interference with IFN signaling through the JAK1/2 inhibitor Ruxolitinib. Patients with a lower *STAT3β/α* mRNA ratio and unfavorable prognosis might benefit from treatment combinations with Ruxolitinib.

## Materials and methods

Additional methods can be found in Supplementary Materials and Methods.

### AML patients

Diagnostic samples (peripheral blood and bone marrow aspirates) from 79 AML patients were collected after written informed consent at the Medical University of Graz (Austria) and processed as described [20]. Only patients receiving curative treatment were included [21]. Patients with FLT3 aberrations, associated with elevated STAT5 signaling, were excluded [22–26]. For patient characteristics see Supplementary Table 1. Publicly available gene expression data sets were analyzed with Kaplan-Meier Plotter [27] (n = 443) and BloodSpot [28] (n = 244).

### Animal studies

Mice were kept under pathogen-free conditions at the Institute of Pharmacology, Medical University of Vienna (Austria). STAT3β-deficient mice were generated by Maritano et al. [11]. For the AML model, fetal liver cells were isolated and retrovirally transfected as described [19]. 2 × 10^5^ Venus^+^ cells were transplanted into 6–8 weeks old male immunocompromised NOD.Cg-Prkdc^scid^Il2rg^tm1Wjl^/SzJ mice (The Jackson Laboratory) *via* tail vein injection.

### Flow cytometry

Unspecific Fc-receptor binding was blocked using an anti-CD16/CD32 antibody (BD Bioscience, Franklin Lakes, NJ, USA). Antibodies (Supplementary Table 3) were added to cell suspension at 1:100–1:200 in PBS containing 2% FBS, incubated for 30 min on ice and protected from light, and afterwards washed twice using PBS. Samples were measured using a Cytoflex S (Beckman Coulter, Brea, California, USA), and data was analyzed with CytExpert (Beckman Coulter) or FlowJo software (TreeStar, Ashland, OR, USA). Living cells were determined according to FSC and SSC and viability was confirmed using 7-AAD staining. Gating was performed using negative controls (untransfected cells or unstained samples) and single-staining controls.

### Colony formation assay

1×10^4^ cells were seeded in 1 ml methylcellulose with recombinant cytokines (SCF, IL-3, IL-6, and EPO) for mouse cells (MethoCult^TM^ GF M3434, Stemcell Technologies, Vancouver, Canada) in 35 mm cell culture dishes and incubated at 37 °C, 95% humidity and 5% CO_2_. After 7 days colonies were counted and harvested for immunophenotyping *via* flow cytometry. For treatments 2 µg/ml IFNAR1 blocking antibody (clone: MAR1-5A3, BioLegend, San Diego, CA, USA), 2 µg/ml IgG isotype control (Invitrogen, Thermo Fisher Scientific, Waltham, MA, USA), 5 µM Ruxolitinib (LC Laboratories, MA, USA) or 0.05% DMSO (Carl Roth, Mannheim, Germany) was supplemented. All images were obtained after 7 days in methylcellulose using an Olympus CKX53 microscope (Olympus, Tokyo, Japan) and an Olympus EP50 camera.

### Statistics

#### Patient data

Stratification in our cohort and in the Kaplan-Meier Plotter into high and low gene expression was performed according to Nagy et al. [29]. Survival analysis was performed using Kaplan-Meier estimates and log-rank (Mantel-Cox) test.

#### Animal and in vitro data

Survival was analyzed using Kaplan-Meier estimates and log-rank (Mantel-Cox) tests. Other data sets were analyzed with unpaired Student’s *t*-*test* using GraphPad Prism. The Shapiro-Wilk test was carried out to test for normal distribution. Outliers were excluded based on Grubbs’ test with significance level alpha = 0.05. Error bars represent means ± SD from at least three independent biological or technical replicates. p-values are indicated as p ≤ 0.05:*, ≤0.01:**, ≤0.001:***, and ≤0.0001:****. Lack of statistical significance was indicated with “ns”.

## Results

### STAT3β-deficient leukemia cells display increased colony formation potential and are less committed to the myeloid lineage

**Fig. 1:**
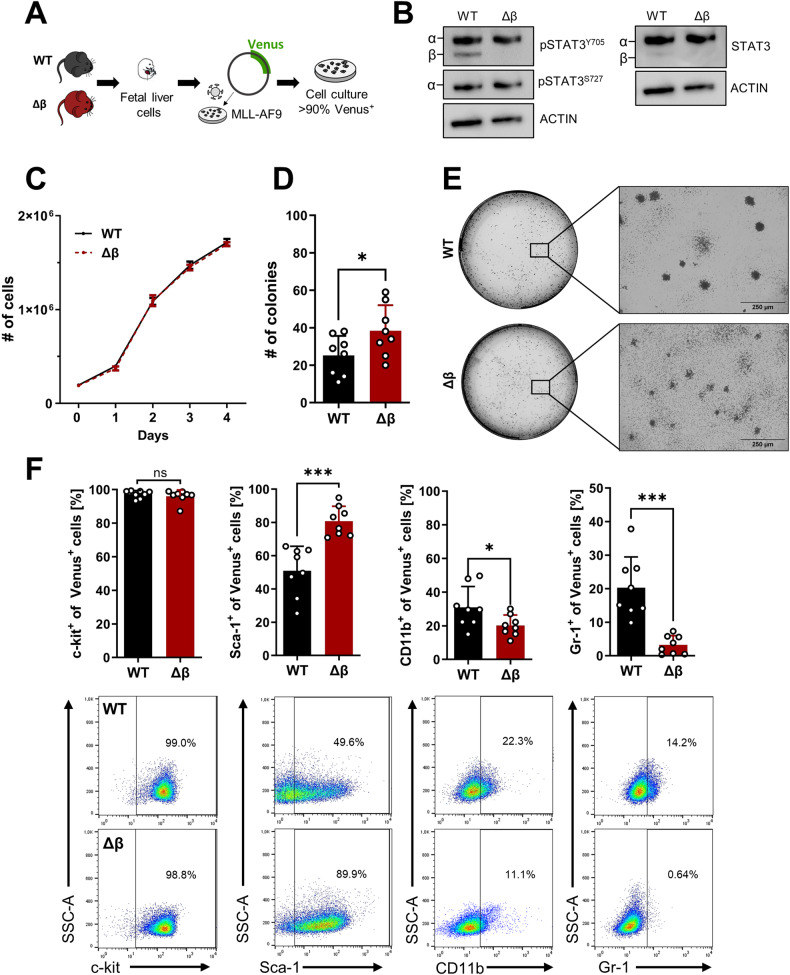
STAT3β-deficient leukemia cells display increased colony formation potential and are less committed to the myeloid lineage. **A** FLCs from WT and STAT3β–deficient (Δβ) mice were retrovirally transformed using a vector encoding *MLL-AF9*. **B** Western blot analysis of *MLL-AF9* transformed FLCs with the indicated antibodies. **C** Growth curves of *MLL-AF9* transformed FLCs (n = 6, 3 independent experiments, 2 cell lines). **D** Colony formation assay of WT and STAT3β-deficient leukemia cells (n = 8, 3 independent experiments with 3 serial replatings). **E** Representative pictures of colonies after 7 days in methylcellulose (40× magnification). **F** Immunophenotyping of leukemia cells after 7 days in methylcellulose *via* flow cytometry and representative dot plots (n = 8, 3 experiments, 3 serial replatings). Statistical analysis was performed using Student’s *t* test and indicated as p ≤ 0.05:*, ≤0.01:**. Error bars represent means ± SD.

To mimic the characteristics of AML blasts, we used an AML mouse model driven by the *MLL-AF9* fusion oncogene that results from the t(9;11)(p22;q23) translocation, which is found in AML patients and associated with an intermediate prognosis [30–32]. Fetal liver cells (FLCs) were isolated from wildtype (WT) and STAT3β-deficient (Δβ) mice and transduced with a retroviral vector encoding *MLL-AF9*, coupled to a Venus fluorescence reporter (Fig. 1A) [11, 33]. The genotype was confirmed *via* PCR (Supplementary Fig. 1A). A similar expression of STAT3α on the mRNA and protein levels (Supplementary Fig. 1B, Fig. 1B) was observed in established homogenous Venus^+^ cells (Supplementary Fig. 1C) that were utilized for all in vitro experiments. Although the absence of STAT3β neither led to a proliferative advantage (Fig. 1C) nor affected cell cycle regulation (Supplementary Fig. 1D) or apoptosis in suspension culture (Supplementary Fig. 1E), STAT3β-deficiency resulted in increased colony formation capacity upon serial replating in methylcellulose (Fig. 1D). Both WT and STAT3β-deficient leukemia cells gave rise to myeloid-type colonies and exhibited similar viability after 7 days (Supplementary Fig. 1F) but displayed a different colony morphology (Fig. 1E, Supplementary Fig. 1G). Immunophenotyping revealed that STAT3β-deficient colonies were comprised of significantly fewer mature myeloid cells, as defined by the myeloid surface markers CD11b and Gr-1, but contained more Sca-1^+^ cells (Fig. 1F), while no discernible differences were observed in suspension culture (Supplementary Fig. 1H). In summary, these experiments demonstrate that loss of STAT3β does not accelerate proliferation but enhances colony forming ability of *MLL-AF9-*transformed FLCs. Furthermore, our findings suggest that STAT3β promotes myeloid differentiation of leukemia cells.

### Leukemia cells lacking STAT3β exhibit reduced differentiation response to G-CSF

**Fig. 2:**
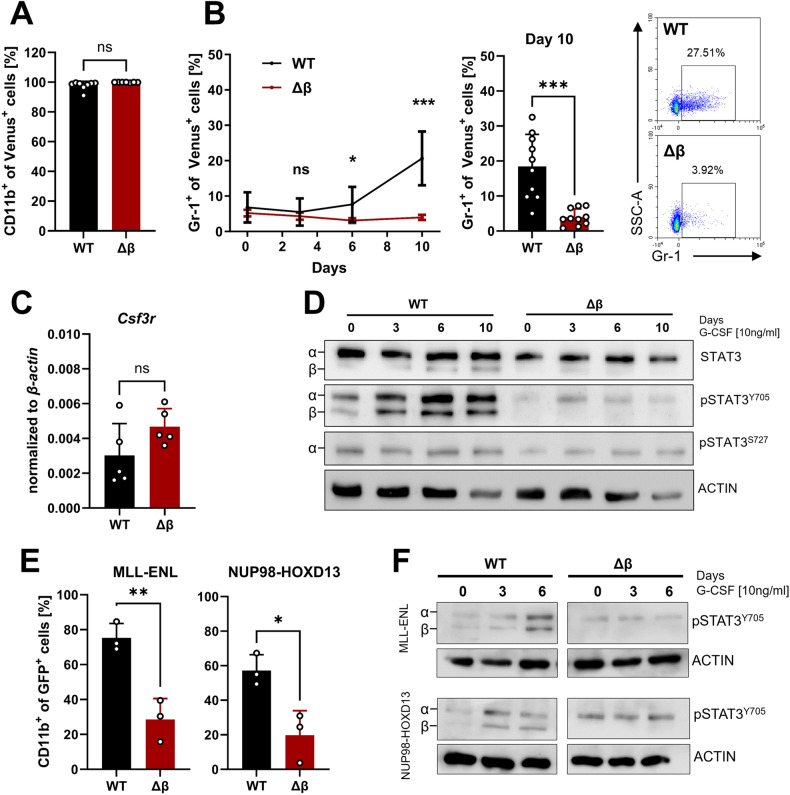
Leukemia cells lacking STAT3β exhibit reduced differentiation response to G-CSF. **A** Flow cytometry quantification of CD11b^+^ cells after 10 days cultured with 10 ng/ml G-CSF. (n = 8, 3 independent experiments, 2–3 cell lines). **B** Quantification of Gr-1^+^ cells after 10 days of G-CSF stimulation *via* flow cytometry and representative dot plots from day 10 (n = 10, 4 independent experiments, 2–3 cell lines). **C** RT-qPCR analysis of G-CSFR (*Csf3r*) expression in suspension culture (n = 5, 3 independent experiments, 2 cell lines). **D** Western blot of G-CSF stimulated samples with the indicated antibodies and time points. **E** Immunophenotyping of *MLL-ENL* and *NUP98-HODX13* transformed FLCs after 10 days G-CSF *via* flow cytometry (n = 3, independent experiments). **F** Western blot of G-CSF stimulated samples (*MLL-ENL* and *NUP98-HOXD13* transformed FLCs) with the indicated antibodies and time points. Statistical analysis was performed using Student’s *t* test and indicated as p ≤ 0.05:*, ≤0.01:**, ≤0.001:***. Error bars represent means ± SD.

To explore the function of STAT3β during differentiation in AML cells, we used granulocyte colony-stimulating factor (G-CSF) as a stimulus. G-CSF regulates the differentiation and function of neutrophils and has been employed in multiple studies investigating the involvement of STAT3 in myeloid differentiation [34, 35]. Since G-CSF stimulation leads to a robust increase in STAT3β phosphorylation after long-term treatment [35, 36], we used G-CSF for up to 10 days to provoke differentiation and STAT3 activation in *MLL-AF9* transformed FLCs. While all cells remained CD11b^+^ (Fig. 2A), solely leukemia cells expressing both STAT3 isoforms responded to G-CSF by notable upregulation of the granulocytic surface marker Gr-1 (Fig. 2B), while on the mRNA level expression of the G-CSF receptor (*Csf3r*) was comparable in suspension culture (Fig. 2C). Furthermore, G-CSF stimulation did not lead to alterations in the expression of the hematopoietic stem cell (HSC) markers c-kit and Sca-1 (Supplementary Fig. 2A). Three days of G-CSF stimulation resulted in a significant increase of STAT3 Y705 phosphorylation only in WT leukemia cells (Fig. 2D, Supplementary Fig. 2B). In contrast, serine 727 (S727) phosphorylation (Fig. 2D) and proliferation were not affected (Supplementary Fig. 2C). This phenomenon is not exclusive to *MLL-AF9* transformed FLCs but was also observed in two additional leukemia models, driven by the fusion genes *MLL-ENL* or *NUP98-HOXD13* [37, 38]. As on these cells Gr-1 was neither detected in suspension culture nor upon stimulation, we used the myeloid marker CD11b as a readout. Upon G-CSF stimulation we detected increased CD11b expression (Fig. 2E) and strong phosphorylation of STAT3 Y705 (Fig. 2F) only when both STAT3 isoforms were present. Similar to our observations in *MLL-AF9*-driven leukemia cells, we did not observe a proliferation advantage in the absence of STAT3β in *MLL-ENL* or *NUP98-HOXD13*-driven leukemia cells (Supplementary Fig. 2D). Taken together, this data demonstrates that STAT3β plays—independent of the driver oncogene—a significant role in orchestrating myeloid differentiation in AML cells as its absence led to impaired myeloid differentiation in response to G-CSF.

### Absence of STAT3β accelerates disease progression in an MLL-AF9-dependent mouse model

**Fig. 3:**
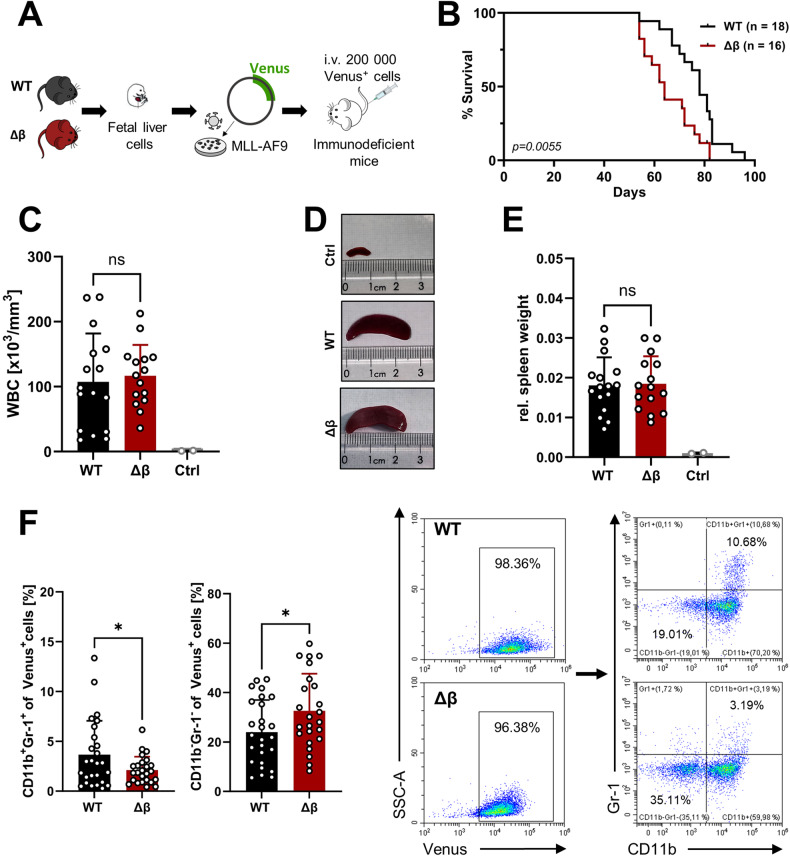
Absence of STAT3β accelerates disease progression in *an MLL-AF9*-dependent mouse model. **A** 200 000 Venus^+^ cells were intravenously (i.v.) transplanted into immunocompromised NOD.Cg-Prkdc^scid^Il2rg^tm1Wjl^/SzJ (NSG) mice. **B** Kaplan-Meier plot of mice receiving STAT3β-deficient or WT leukemia cells (3 independent experiments, median survival: WT = 78 days; Δβ = 64 days). Log-rank (Mantel-Cox) test was performed to analyze the survival difference between the two groups (P = 0.0055). **C** WBC count of diseased animals at the time point of euthanasia (n = 15). **D** Representative pictures of spleens from diseased animals. **E** Spleen-to-body weight of diseased animals (n = 15, ctrl: n = 2). **F** Flow cytometry quantification of myeloid markers on leukemia cells isolated from the BM and SP of each animal (n = 24, BM and SP pooled from 12 animals) and representative dot plots. Further statistical analysis was performed using Student’s *t* test and indicated as p ≤ 0.05:*. Error bars represent means ± SD. (WBC white blood cell).

We next intravenously transplanted *MLL-AF9* transformed WT or STAT3β-deficient FLCs into immunocompromised NSG mice and monitored disease progression *via* white blood cell counts (Fig. 3A, Supplementary Fig. 3A). Animals transplanted with STAT3β-deficient leukemia cells exhibited significantly shorter OS (Fig. 3B) compared to mice receiving WT leukemia cells demonstrating its pivotal role as a tumor suppressor. At the endpoint no significant differences were observed between recipients of *MLL-AF9* transformed WT or STAT3β-deficient cells in leukemia infiltration of the bone marrow (BM) and spleen (SP) (Supplementary Fig. 3B), blasts detected in the peripheral blood (Fig. 3C, Supplementary Fig. 3C), spleen size (Fig. 3D), or ex vivo proliferation (Supplementary Fig. 3D). In contrast, and consistent with our in vitro data, blasts lacking STAT3β were less committed to the myeloid lineage (CD11b^+^ Gr-1^+^) and comprise more CD11b^-^Gr-1^-^ cells suggesting a more immature state in blasts lacking STAT3β (Fig. 3F). Together, these data indicate that the absence of STAT3β in blasts accelerates AML development and restricts myeloid differentiation in vivo, underscoring its role as a tumor suppressor.

### Gene expression analysis reveals enrichment of IFN-regulated genes in blasts lacking STAT3β

**Fig. 4:**
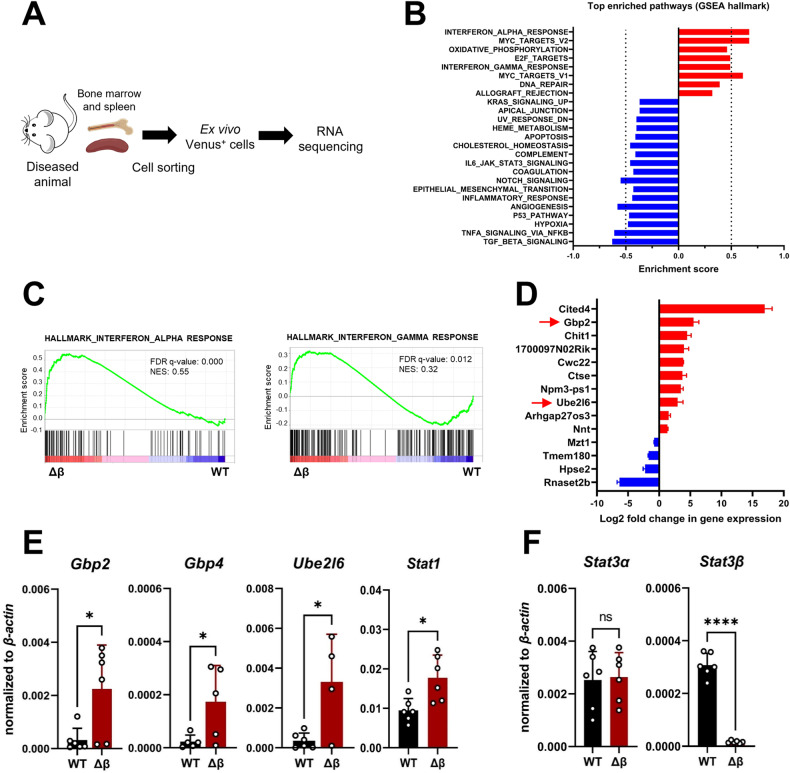
Gene expression analysis reveals enrichment of IFN-regulated genes in blasts lacking STAT3β isolated from diseased animals. **A** Sample preparation for RNA sequencing (n = 3 per group and organ). For sorting strategy see Supplementary Fig. 4B. **B** Top enriched pathways (nominal p < 1%) in blasts lacking STAT3β. **C** GSEA plots of WT and STAT3β-deficient blasts isolated from SP of diseased animals (FDR False discovery rate, NES normalized enrichment score). **D** Significant hits shared between BM- and SP-derived blasts (n = 6). **E** RT-qPCR of ex vivo samples (n = 6, pooled BM and SP). **F** RT-qPCR on *Stat3* isoform expression in ex vivo samples from BM and SP (n = 6, pooled). Statistical analysis was performed using Student’s *t* test and indicated as p ≤ 0.05:*, ≤0.01:**, ≤0.001:*** and ≤0.0001:****. Error bars represent means ± SD.

To identify STAT3β-regulated processes or target genes that affect disease outcome, blasts were isolated from the BM and SP of diseased animals, sorted based on the co-expressed fluorophore Venus, and subjected to RNA sequencing (Fig. 4A, Supplementary Fig. 4A, B). To assess which cellular processes are dysregulated due to STAT3β-deficiency, we performed gene set enrichment analysis (GSEA). An enrichment of genes involved in IFN response, Myc targets, and oxidative phosphorylation was observed in the absence of STAT3β (Fig. 4B). In line with previous reports [2, 10], pathways related to hypoxia, inflammatory response, and IL6-JAK-STAT3 signaling were reduced in STAT3β-deficient blasts (Fig. 4B, Supplementary Fig. 4C). KEGG pathway analysis revealed that genes that were differentially expressed in the absence of STAT3β were enriched in the processes “hematopoietic cell lineage” and “cytokine-receptor interaction” (Supplementary Fig. 4D). This is in accordance with the observation that loss of STAT3β reduces myeloid differentiation of leukemia cells both in vitro and in vivo (Figs. 1F, 3F). GSEA revealed that a strong enrichment in gene expression in STAT3β-deficient blasts was observed in genes involved in type I (IFNα/β) and type II (IFNγ) IFN signaling (Fig. 4C). By overlapping the significantly up- and downregulated genes between BM- and SP-derived blasts, we found *Gbp2* and *Ube2l6*, which are directly involved in IFN signaling, upregulated in the absence of STAT3β (Fig. 4D). Additionally, *Gbp4* and *Stat1* were among the highest upregulated genes in GSEA data (Supplementary Fig. 4E). RT-qPCR confirmed a threefold increase in *Ube2l6* and *Gbp2* expression and a twofold increase in *Stat1* expression, in STAT3β-deficient blasts (Fig. 4E). Moreover, *Stat3α* expression was comparable between blasts isolated from BM and SP confirming that the observed changes in gene expression are attributed to STAT3β rather than STAT3α (Fig. 4F). Taken together, this data suggests that STAT3β represses IFN response in AML cells.

### STAT3β deficiency increases IFN responsiveness

**Fig. 5:**
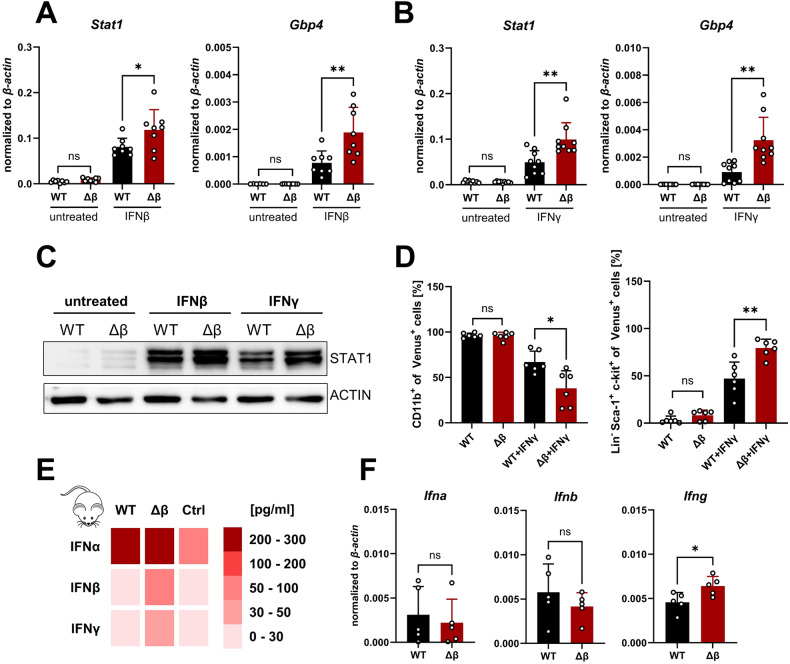
STAT3β deficiency increases IFN responsiveness. **A** mRNA expression after 3 h 100 U/ml IFNβ stimulation (n = 8, 3 independent experiments, 2–3 cell lines). **B** mRNA expression after 3 h 100 ng/ml IFNγ stimulation (n = 9, 3 independent experiments, 3 cell lines). **C** Western blot after 24 h IFN treatment. **D** Flow cytometry quantification of CD11b^+^ and Sca-1^+^ cells after 48 h IFNγ treatment (n = 6, 3 independent experiments, 2 cell lines). **E** Heatmap represents the average concentration measured in the plasma of diseased animals *via* multiplex assay. (n = 5 per group, 3 controls). **F** mRNA expression of *Ifna, Ifnb,* and *Ifng* in leukemia cells after 7 days in methylcellulose analyzed *via* RT-qPCR (n = 5, 3 independent experiments, 2 cell lines). Statistical analysis was performed using Student’s *t* test and indicated as p ≤ 0.05:*, ≤0.01:**. Error bars represent means ± SD.

To investigate whether the IFN response depends on STAT3 isoform expression, we stimulated *MLL-AF9* transformed FLCs for 3 h with either IFNβ or IFNγ. While no differences occurred in the naïve state, both agents caused a significantly stronger increase in the expression of IFN-inducible genes (*Gbp4, Stat1*) in STAT3β-deficient compared to WT leukemia cells (Fig. 5A, B). These differences were not caused by altered levels of interferon alpha/beta receptor 1 (IFNAR1) expression between genotypes (Supplementary Fig. 5A). After a 24 h stimulation with either IFNβ or IFNγ, we observed increased levels of STAT1 protein in both genotypes. Interestingly, a higher expression was observed when STAT3β was absent (Fig. 5C, Supplementary Fig. 5B). In healthy HSCs, IFNγ was shown to modulate lineage-specific myeloid differentiation [39] and to promote the expansion of Lin^-^Sca-1^+^c-kit^+^ (LSK) cells potentially through upregulation of Sca-1 *via* JAK/STAT signaling [40, 41]. To assess the potential impact of IFNγ on the differentiation state of AML cells, we treated the cells for 48 h with IFNγ followed by immunophenotyping. Strikingly and in line with STAT3β-deficient leukemia cells displaying attenuated commitment to the myeloid lineage, a significant decrease in CD11b expression and an increase of LSK blasts was detected upon IFNγ stimulation, which was stronger in leukemia cells lacking STAT3β (Fig. 5D, Supplementary Fig. 5C). These differences were independent of effects on cell viability (Supplementary Fig. 5D).

To investigate whether increased expression of IFN-inducible genes is linked to elevated cytokine production, we analyzed IFN levels in the plasma of leukemic mice. Remarkably, elevated levels of IFNα, IFNβ, and IFNγ were detected in comparison to healthy controls (Fig. 5E). While high IFNα concentrations were found in both groups, we observed higher IFNβ and IFNγ levels in the absence of STAT3β. In line with previous work demonstrating AML blasts themselves are capable of releasing IFNγ, associated with poorer OS [42], we detected *Ifna, Ifnb* and *Ifng* expression after 7 days in methylcellulose, with a significantly higher *Ifng* expression in colonies formed by STAT3β-deficient leukemia cells (Fig. 5F). In summary, these data indicate that loss of STAT3β leads to enhanced IFN signaling, and potentially increased IFN production in leukemia cells.

### Interference with IFN signaling is a vulnerability of STAT3β-deficient leukemia cells

**Fig. 6:**
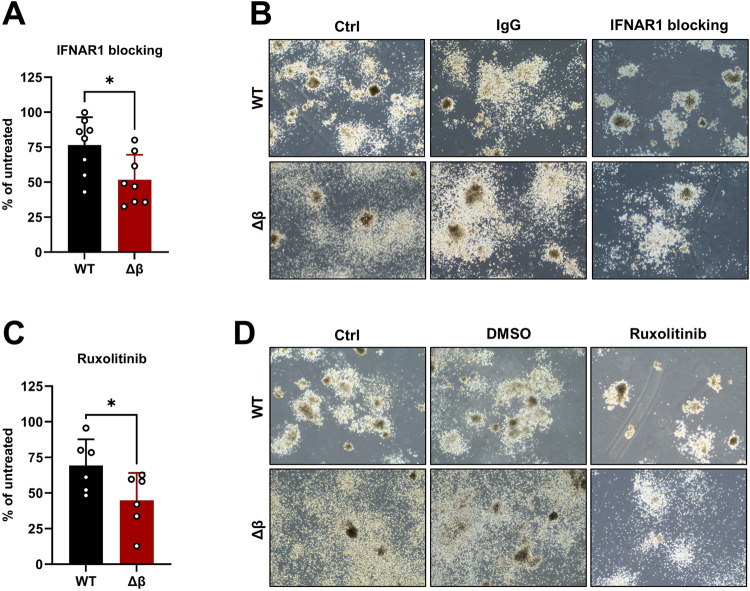
Interference with IFN signaling is a vulnerability of STAT3β-deficient cells. **A** Colonies formed in the presence of 2 µg/ml IFNAR1 blocking antibody normalized to untreated (n = 8 from 3 independent experiments, 3 cell lines). **B** Representative images of colonies formed after 7 days in methylcellulose ± IFNAR1 blocking antibody or IgG control. **C** Colonies formed in the presence of 5 µM Ruxolitinib normalized to untreated (n = 6, 3 independent experiments, 2 cell lines). **D** Representative images of colonies formed ± Ruxolitinib or DMSO control. Statistical analysis was performed using Student’s *t* test and indicated as p ≤ 0.05:*. Error bars represent means ± SD.

Thus far, our data indicated that STAT3β-deficient leukemia cells induced disease at a significantly accelerated rate compared to WT cells (Fig. 3B), accompanied by enhanced IFN signaling (Fig. 4C). This suggests that increased expression of IFN-inducible genes may contribute to an adverse disease outcome. As we detected significantly higher IFN levels in the plasma of leukemic mice (Fig. 5E) and IFN-inducible genes in colonies, particularly in the absence of STAT3β (Supplementary Fig. 6A), we hypothesized that IFN signaling might contribute to the leukemogenic potential of AML blasts. To assess whether interference with type I IFN signaling could ameliorate disease outcomes, we conducted colony formation assays (CFAs) in the presence and absence of an IFNAR1-blocking antibody. Indeed, we observed a substantial reduction of colony numbers after 7 days in methylcellulose for both WT and STAT3β-lacking leukemia cells with a stronger impact on the latter (Fig. 6A, B, Supplementary Fig. 6B, D). Accordingly, IFNAR1 blocking reduced IFN-inducible genes (*Stat1, Gbp4, Ube2l6*), in both groups (Supplementary Fig. 6C). To inhibit both type I and type II IFN signaling, we tested the JAK1/2 inhibitor Ruxolitinib. As expected, treatment with Ruxolitinib was sufficient to completely inhibit IFNγ-induced upregulation of *Stat1* and *Gbp2* in *MLL-AF9* transformed FLCs (Supplementary Fig. 6E). Furthermore, Ruxolitinib treatment resulted in a significant reduction in colony numbers of STAT3β-deficient leukemia cells compared to WT cells (Fig. 6C, D, Supplementary Fig. 6F). To conclude, this data suggests that blockade of IFN signaling is a STAT3β-isoform specific vulnerability of leukemia cells, which could be of therapeutic relevance.

### High expression of STAT1/IFN-inducible genes is associated with low STAT3β expression and with adverse outcomes in patients with AML

**Fig. 7:**
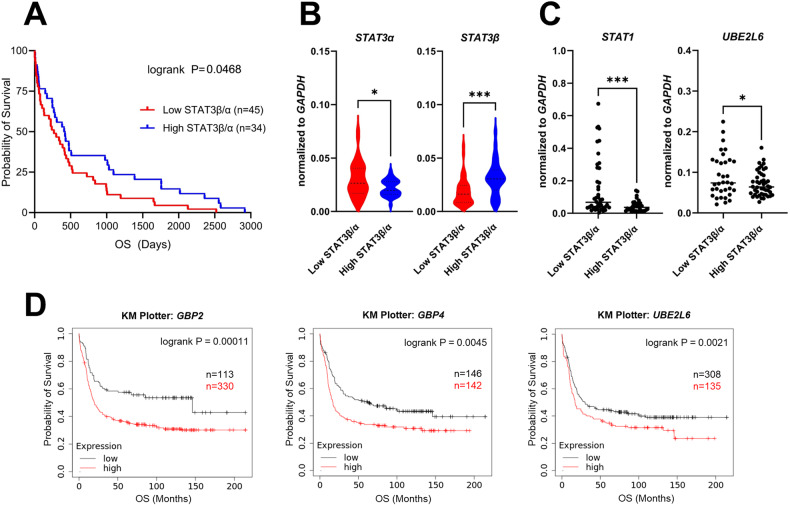
High expression of STAT1/IFN-inducible genes is associated with low *STAT3β* expression and adverse outcomes in AML patients. **A** RT-qPCR analysis of STAT3 isoform expression in 79 AML patient samples. A low *STAT3β/α* mRNA ratio correlates with poor OS. Patients were stratified according to the best cut-off value. Statistical analysis was performed using log-rank (Mantel-Cox) test. **B** RT-qPCR of *STAT3α* and *STAT3β* (normalized to *GAPDH*) in patients with a low or high *STAT3β/α* ratio (n = 79). Data were compared using the Student’s *t* test. **C** *STAT1* and *UBE2L6* expression in patients with a low or high *STAT3β/α* ratio (n = 79). Data were compared using the Student’s *t* test. **D** KM Plotter analysis of RNA sequencing data of AML patients showing a correlation between high expression of *GBP2, GPB4*, and *UBE2L6* with OS. Patients were stratified according to the best cut-off value. (kmplot.com), status: 15.01.2024. Statistical analysis was performed using log-rank (Mantel-Cox) test. p ≤ 0.05:*, ≤0.01:**, ≤0.001:***.

As our results indicate a link between IFN signaling and disease outcome, we explored the relevance of these findings in the clinical context. To validate the association between high expression of IFN-inducible genes and imbalanced STAT1/3 signaling, we accessed a patient cohort comprising 79 newly diagnosed AML patients (Supplementary Table 1). In line with our previous study, we confirmed in this new cohort *via* RT-qPCR that a low *STAT3β/α* mRNA ratio correlates with adverse disease outcomes (Fig. 7A) which is independent of age and gender of the patients (Supplementary Fig. 7A, B). Notably, *STAT3β* levels were primarily responsible for a lower *STAT3β/α* ratio, while *STAT3α* expression appears to be of less relevance (Fig. 7B). Intriguingly, patients with low *STAT3β/α* ratio displayed a significantly higher *STAT1* and *UBE2L6* gene expression (Fig. 7C). In this cohort we noted a trend suggesting higher expression of STAT1 and IFN-inducible genes is potentially associated with adverse disease outcomes (Supplementary Fig. 7C) We therefore tested this in a larger patient cohort using two different publicly available RNA sequencing data sets of AML patients (KM Plotter: Affymetrix, BloodSpot: TCGA), and correlated the expression of IFN-inducible genes to OS. Remarkably, high expression of *GBP2* and *UBE2L6* correlated with significantly poorer OS in both cohorts (Fig. 7D, Supplementary Fig. 7D). To conclude, we here describe a specific contribution of STAT3β in fine-tuning IFN response by repressing STAT1-mediated IFN signaling in AML blasts, which improves disease outcome.

## Discussion

We previously demonstrated that the *STAT3β/α* mRNA ratio serves as a prognostic marker, correlating with disease outcomes in AML patients [19]. Here, we provide strong evidence for STAT3β acting as a tumor suppressor in AML and uncovered the underlying mechanism behind its protective role. The absence of STAT3β in AML cells led to impaired G-CSF-induced myeloid differentiation and STAT3α phosphorylation in vitro. Using *an MLL-AF9*-driven AML mouse model we confirmed a reduced commitment to the myeloid lineage in leukemia cells in vivo. The imbalance of STAT3 isoforms in leukemia cells resulted in enhanced IFN signaling in the absence of STAT3β together with shorter survival of leukemic mice. Intriguingly, we confirmed that higher expression of IFN-inducible genes correlates with shorter OS in AML patients.

The impact of STAT3 on regulating the balance between self-renewal and differentiation of HSCs is highly dependent on the type of hematopoietic progenitor and the stimulus. Especially during myeloid differentiation in response to G-CSF, robust and sustained STAT3 activation is crucial for differentiation and survival of myeloid progenitors [43–45]. Several studies reported increased STAT3β activity upon G-CSF-induced myeloid differentiation in healthy but also transformed leukemia cells [34, 35]. Accordingly, phosphorylation of STAT3 in response to G-CSF in murine leukemia cells only occurred if both isoforms were present. Interestingly, phosphorylation of STAT3α in STAT3β-deficient blasts also remained absent, together with impaired myeloid differentiation. These findings indicate that STAT3β is pivotal for a G-CSF-induced myeloid differentiation program in AML blasts. As we did not observe alterations in G-CSF receptor (*Csf3r*) mRNA expression or in proliferation upon G-CSF stimulation, we assume that the absence of STAT3β does not affect receptor expression per se, but rather impairs endosomal recycling of the receptor causing reduced signaling, as previously suggested [46–48]. However, to obtain more detailed mechanistic insights further research is required. In vivo, we also identified a reduction in myeloid lineage commitment in blasts lacking STAT3β, together with more rapid disease progression. Consistent with this, pediatric AML patient samples with low STAT3 phosphorylation upon G-CSF stimulation showed poorer OS than patients with a stronger STAT3 response [46].

As anticipated, the absence of STAT3β led to accelerated disease progression, together with enhanced IFN signaling in vivo. Since IFN signaling plays a central role in cancer progression by promoting inflammation and reprogramming the tumor microenvironment, but also in multiple anti-tumor properties, its effects are highly context-dependent [49–51]. In AML patients, IFN signatures have been reported to correlate with favorable prognosis [52, 53], and negative outcomes as well [42]. One possible explanation for these contradictory results could be a concentration-dependent effect of IFN leading to either an acute or chronic response [51, 54]. While we observed high IFNα concentrations in the plasma of diseased animals in both groups, we detected IFNβ and IFNγ to a higher extent in the absence of STAT3β. Constant production of IFN at a low level, also known as tonic signaling [55], was reported for AML blasts leading to rearrangement of the tumor microenvironment driving leukemogenesis [42]. Accordingly, we observed that blocking type I IFN signaling leads to impaired colony formation ability, particularly in the absence of STAT3β. Furthermore, interference with both type I and type II IFN signaling with Ruxolitinib resulted in significantly decreased colony formation of STAT3β-deficient blasts. Ruxolitinib, approved for myeloproliferative neoplasms, was tested in clinical trials including patients with secondary AML or relapsed patients with moderate effectiveness [56].

Since STAT1 expression was elevated in STAT3β-deficient leukemia cells along with IFN-inducible genes, we postulate that the enhanced IFN signaling results from an imbalance in STAT1/3 signaling due to the absence of STAT3β. While STAT1 and STAT3 activate transcription of an overlapping set of genes, they do not bind to identical DNA elements, which is likely to account for their distinct biological effects [57]. In line, the balance between STAT1/STAT3 activation in response to IFN determines the inflammatory potency in healthy but also transformed cells [58, 59]. IFNβ and IFNγ stimulation in the absence of STAT3β in vitro revealed significantly higher expression of STAT1 as well as other IFN-inducible genes. In accordance, STAT3 has been described to attenuate STAT1-mediated IFN signaling [58, 59]. However, in our setting, loss of STAT3β alone was sufficient to enhance STAT1 expression in AML blasts. To ascertain the clinical relevance, we analyzed 79 AML patient samples. *STAT1* and the IFN-inducible gene *UBE2L6* were significantly higher expressed in patients with a low *STAT3β/α* mRNA ratio. Using two distinct publicly available RNA sequencing data sets of AML patients, we confirmed a correlation between higher expression of a set of IFN-inducible genes with poorer OS.

To conclude, in this study, we gained novel insights into the protective role of STAT3β in AML. STAT3β favors myeloid differentiation and fine-tunes tonic IFN signaling in experimental mice and human AML patients. We here describe for the first time STAT3β as a repressor of IFN signaling in AML blasts by maintaining the balance of STAT1-mediated IFN signaling. Thus, using the *STAT3β/α* mRNA ratio as a prognostic marker could hold crucial information for targeted treatment approaches. Specifically, patients exhibiting a low *STAT3β/α* ratio could benefit from therapeutic interventions targeting STAT1/IFN signaling.

### Supplementary information

Supplementary Material

Original Western Blots

## Data Availability

RNA sequencing data is available *via* Gene Expression Omnibus (GEO) database under accession number GSE261198.
