# Supplementary material for: A novel function of STAT3β in suppressing interferon response improves outcome in acute myeloid leukemia
Source: Cell Death Dis. 2024 May 28;15(5):369. doi: 10.1038/s41419-024-06749-9 (PMC11133483; doi:10.1038/s41419-024-06749-9)
Supplement: Supplementary file 1 — Supplementary Material [file 41419_2024_6749_MOESM1_ESM.pdf]

“A novel function of STAT3β in suppressing interferon response improves outcome in acute myeloid leukemia” by Edtmayer et al.

Supplementary materials and methods

Genotyping PCR

DNA was isolated from tissue samples of mouse offspring or cell pellets using the Extracta™ DNA prep for PCR for tissue (Quantabio, Beverly, MA, USA) followed by PCR using OneTaq® Quick-Load 2x Master Mix with Standard Buffer (New England BioLabs, Ipswich, MA, USA) according to the manufacturer’s protocol. Primer sequences are listed in **Suppl. Table 1**.

RNA isolation and RT-qPCR

RNA was isolated using QIAzol Lysis Reagent (Qiagen, Hilden, Germany) or for patient samples using the RNeasy Mini Kit (Qiagen). Reverse transcription was performed using RevertAid H Minus First Strand cDNA Synthesis Kit (Thermo Fisher Scientific, Waltham, MA, USA) according to the manufacturer’s instructions. Reverse transcription-quantitative polymerase chain reaction (RT-qPCR) was performed in triplicates using SsoAdvanced™ Universal SYBR® Green Supermix (Bio-rad, Hercules, CA, USA) and measured on a qTower^3 (Analytik Jena, Jena, Germany). Readings were normalized to *β-actin* (mouse) or *GAPDH* (human) and quantified as described.(1) Primer sequences are listed in **Suppl. Table 1**.

Western blot

$1x10^5$ cells were lysed in 10µl 1x Laemmli sample buffer (Bio-rad) and incubation for 20min at 95°C. Equal amounts of protein were loaded on a 10% SDS-polyacrylamide gel and blotted onto a 0.45µm polyvinylidene fluoride (PVDF) membrane (Thermo Fisher Scientific). After blocking with 5% milk in phosphate-buffered saline (PBS) supplemented with 0.1% Tween20 (PBS-T), membranes were incubated in primary antibody (**Suppl. Table 2**) diluted in 5% bovine serum albumin (BSA) in PBS-T overnight at 4°C. After washing, membranes were incubated in 5% BSA in PBS-T containing the HRP-linked secondary antibody (**Suppl. Table 2**). For detection, SuperSignal™ West Femto Maximum Sensitivity Substrate (Thermo Fisher Scientific) was added and imaged using a ChemiDoc imager (Bio-rad). Protein expression was quantified using ImageJ (version 1.53t) and normalized to ACTIN. Original full length uncropped Western blots can be found in the Supplemental Material.

Blood analysis and organ processing

Peripheral blood was collected *via* punction of the *vena facialis* in EDTA tubes and analyzed using a blood counter (Scil Vet ABC, Gurnee, IL, USA). Peripheral blood smears were stained using an H&E fast staining kit (Carl Roth, Mannheim, Germany). Plasma was collected by

centrifugation for 15min at 2,000g and 4°C. Supernatant was collected and stored at -80°C until further usage. Spleens were isolated and mashed through a 70µm cell strainer followed by red blood cell lysis. Bone marrow cells were harvested from hind legs. Samples were cryopreserved as single cells suspension in FBS supplemented with 10% DMSO and stored at -80°C until needed. For flow cytometry analysis the samples were thawed in PBS, stained, and analyzed simultaneously.

RNA sequencing

Leukemic blasts were isolated from bone marrow and spleen of diseased animals by sorting Venus$^+$ cells using a BD FACSymphony^{TM} S6 Cell Sorter (BD Bioscience) and analyzed using BD FACSDiva Software. RNA was isolated using RNeasy Mini Kit (Qiagen) and sequenced with an Illumina HiSeq3000/4000 sequencer using 50bp single-end libraries. Quality checks of the raw sequence reads were done with FastQC(2) (version 0.11.9). Then we applied PRINSEQ-lite(3) (version 0.20.4) for quality based read filtering and trimming. The remaining high-quality reads were aligned against the mouse reference genome (GRCm38) using STAR(4) (version 2.5.0b) and post processed with samtools(5) (version 1.4). Read counts were obtained with featureCounts(6) (version 2.0.3) and for normalization and differential gene expression analysis we applied DESeq2(7) (version 1.40.2). GSEA (GSEA version 4.2.2) was done with genes that show an adjusted p-value < 0.05 according to the provider's protocol.(8) Gene sets were downloaded from the Molecular Signature Database (http://software.broadinstitute.org/gsea/msigdb/index.jsp) and permutations were set to 1,000.

Multiplex immunoassay

Cytokine levels were measured in plasma of diseased animals (1:2 dilution) using the ProcartaPlex^{TM} Mouse and Rat Mix & Match panels (Invitrogen, Thermo Fisher Scientific) according to the manufacturer’s protocol. All measurements were performed using a Bio-Plex 200 System (Bio-rad) following the manufacturer’s instructions.

Retroviral infection

Platinum-E packaging cells were transfected with the *pMSCV-MLL-AF9-IRES-Venus* construct, which was a kind gift from Johannes Zuber (Research Institute of Molecular Pathology, Vienna, Austria), by calcium phosphate co-precipitation. FLCs were thawed and cultured one day prior to infection and spinoculated at 1,000g for 90min three times on two consecutive days with retroviral supernatant supplemented with 10µg/ml polybrene (Sigma-Aldrich, St. Louis, MO, USA). Two days after the first infection $2x10^5$ MLL-AF9/Venus$^+$ cells were transplanted into 6 to 8 weeks old male immunocompromised NOD.Cg-Prkdc^{scid}Il2rg^{tm1Wjl}/SzJ mice (The Jackson Laboratory, Bar Harbor, ME, USA) *via* tail vein injection. The remaining cells were cultured until homogenous Venus$^+$ cells were obtained.

Similarly, the two other AML cell lines were generated using the *pMSCV-FlagMLL-pI-ENL* construct (Addgene plasmid #20873),(9) which was a gift from Robert Slany (University of Erlangen, Bavaria, Germany) and the *pMSCV-NUP98-HOXD13-IRES-GFP* construct provided by Rotraud Wieser (Medical University of Vienna, Vienna, Austria).

Cell culture

Unless otherwise stated, all cell culture reagents were purchased from Gibco (Thermo Fisher Scientific). All cells were maintained under 37°C, 95% humidity and 5% CO_2. Platinum-E packaging cells were a kind gift of Florian Grebien (University of Veterinary Medicine Vienna, Vienna, Austria) and cultured in DMEM supplemented with 10% FBS, 100U/ml penicillin/streptomycin, 2mM L-glutamine. Murine primary cells were cultured in DMEM and IMDM (1:1) supplemented with 10% FBS, 100U/ml penicillin/streptomycin, 4mM L-glutamine, 50µM β-mercaptoethanol, 10ng/ml hIL-6 (Immunotools GmbH, Friesoythe, Germany), 10ng/ml mIL-3 (ImmunoTools GmbH) and 100ng/ml mSCF (generated in-house). *Ex vivo* cells were established by continuous culture of AML blasts isolated from bone marrow and spleen of diseased animals as described.(10) Experiments with *ex vivo* cells were performed in RPMI supplemented with 10% FBS, 100U/ml penicillin/streptomycin, 4mM L-glutamine, 100ng/ml mSCF. All cells were regularly tested for mycoplasma contamination using the MycoAlert Detection Kit (Lonza, Verviers, Belgium) according to the manufacturer’s instructions. For granulocyte-colony stimulating factor (G-CSF) stimulation cell culture medium was supplemented with 10ng/ml G-CSF (PeproTech, Rocky Hill, NJ, USA) and refreshed every two to three days. For all interferon treatments 100U/ml recombinant mouse IFNβ (Sigma-Aldrich) or 100ng/ml recombinant mouse IFNγ (Immunotools GmbH) was supplemented.

Growth curves

1.5×10^5 cells were seeded in triplicates in 12-well plates containing 1ml cell culture medium on day 0. For up to 4 days cell numbers were measured using a Cytoflex S (Beckman Coulter).

Cell cycle and apoptosis staining

For cell cycle analysis, cells were fixed using ice-cold 70% ethanol and stained with 5µl 7AAD (eBioscience, Thermo Fisher Scientific) followed by flow cytometry analysis. For apoptosis analysis, cells were fixed using Annexin V Binding Buffer (BioLegend, San Diego, CA, USA), followed by staining with 5µl Annexin V (BioLegend) and 5µl 7AAD in 100µl Annexin V Binding Buffer. All samples were analyzed using a Cytoflex S (Beckman Coulter).

Supplementary References

1. Livak KJ, Schmittgen TD. Analysis of Relative Gene Expression Data Using Real-Time Quantitative PCR and the 2−ΔΔCT Method. *Methods* **25**, 402-408 (2001).
2. Babraham Bioinformatics: FastQC A Quality Control tool for High Throughput Sequence Data. 2024. https://www.bioinformatics.babraham.ac.uk/projects/fastqc/.
3. Schmieder R, Edwards R. Quality control and preprocessing of metagenomic datasets. *Bioinformatics* **27**, 863-864 (2011).
4. Dobin A, Davis CA, Schlesinger F, Drenkow J, Zaleski C, Jha S, et al. STAR: ultrafast universal RNA-seq aligner. *Bioinformatics* **29**, 15-21 (2013).
5. Li H, Handsaker B, Wysoker A, Fennell T, Ruan J, Homer N, et al. The Sequence Alignment/Map format and SAMtools. *Bioinformatics* **25**, 2078 (2009).
6. Liao Y, Smyth GK, Shi W. featureCounts: an efficient general purpose program for assigning sequence reads to genomic features. *Bioinformatics* **30**, 923-930 (2014).
7. Love MI, Huber W, Anders S. Moderated estimation of fold change and dispersion for RNA-seq data with DESeq2. *Genome Biol* **15**, 1–21 (2014).
8. Subramanian A, Tamayo P, Mootha VK, Mukherjee S, Ebert BL, Gillette MA, et al. Gene set enrichment analysis: A knowledge-based approach for interpreting genome-wide expression profiles. *Proc Natl Acad Sci U S A* **102**, 15545–15550 (2005).
9. Bach C, Mueller D, Buhl S, Garcia-Cuellar MP, Slany RK. Alterations of the CxxC domain preclude oncogenic activation of mixed-lineage leukemia 2. *Oncogene* **28**, 815–823 (2009).
10. Schmoellerl J, Barbosa IAM, Eder T, Brandstoetter T, Schmidt L, Maurer B, et al. CDK6 is an essential direct target of NUP98 fusion proteins in acute myeloid leukemia. *Blood* **136**, 387-400 (2020).

Supplementary Table 1: Patient characteristics.

Patient characteristics	
**Total number of patients**	79
Males	50 (63.3%)
Females	29 (36.7%)
**Age (years)**	
Median	66
Range	23-89
**WBC at diagnosis (10^9/l)**	
Median	6
Range	0-388
**BM blasts at diagnosis (%)**	
Median	68
Range	15-100
**Pathogenic/likely pathogenic mutations (number of samples)**	
KRAS	12
NRAS	14
RUNX1	13
IDH2	8
WT1	2
ASXL1	14
TET2	24
DNMT3A	13
NPM1	11
CEBPA	13
TP53	17
FLT3 / FLT3-IDH	0
Other Mutations (IDH1, CRSF2, STAG2, KIT, etc.)	48
**Therapy**	
Intensive incl. allogeneic hematopoietic stem cell transplantation	45
Non-intensive	28
Best supportive care	6

Supplementary Table 2: List of primer sequences used for genotyping PCR and RT-qPCR.

Gene	Primer sequence (5’ → 3’)	Method
Stat3 WT forward[11]	TGGCTGGCAAGGGCTTCTCCTT	Genotyping PCR
Stat3 WT reverse[11]	TGCCAGTGGCCAGCAGGTGGA	Genotyping PCR
Stat3Δαβ forward[11]	CAGGTCCTGCCCCGTACCTGA	Genotyping PCR
Stat3Δαβ reverse[11]	CTGCATTCTAGTTGTGGTTTGTCC	Genotyping PCR
Stat3α forward	GTCTACCTCTACCCCGACATTCC	RT-qPCR (mouse)
Stat3α reverse	CATCGGCAGGTCAATGGTATTGC	RT-qPCR (mouse)
Stat3β forward	GTCTACCTCTACCCCGACATTCC	RT-qPCR (mouse)
Stat3β reverse	CCAAACTGCATCAATGAATGGTGT	RT-qPCR (mouse)
β-actin forward	GCTCATAGCTCTTCTCCAGGG	RT-qPCR (mouse)
β-actin reserve	CCTGAACCCTAAGGCCAACCG	RT-qPCR (mouse)
Csf3r forward	CTGATCTTCTTGCTACTCCCCA	RT-qPCR (mouse)
Csf3r reverse	GGTGTAGTTCAAGTGAGGCAG	RT-qPCR (mouse)
Gbp2 forward	CTGCACTATGTGACGGAGCTA	RT-qPCR (mouse)
Gbp2 reverse	GAGTCCACACAAAGGTTGGAAA	RT-qPCR (mouse)
Gbp4 forward	GGAGAAGCTAACGAAGGAACAA	RT-qPCR (mouse)
Gbp4 reserve	TTCCACAAGGGAATCACCATTTT	RT-qPCR (mouse)
Ube2l6 forward	GTGGCGAAAGAGCTGGAGAG	RT-qPCR (mouse)
Ube2l6 reverse	GGGGAAATCAATCCGCACTTG	RT-qPCR (mouse)
Stat1 forward	TCACAGTGGTTCGAGCTTCAG	RT-qPCR (mouse)
Stat1 reverse	GCAAACGAGACATCATAGGCA	RT-qPCR (mouse)
Ifna forward	ATGGCTAGGCCCTTTGCTTTC	RT-qPCR (mouse)
Ifna reverse	CAGTTCCTTCATCCCGACCAG	RT-qPCR (mouse)
Ifnb forward	CAGCTCCAAGAAAGGACGAAC	RT-qPCR (mouse)
Ifnb reverse	GGCAGTGTAACTCTTCTGCAT	RT-qPCR (mouse)
Ifng forward	AAGTGGCATAGATGTGGAAG	RT-qPCR (mouse)
Ifng reverse	GAATGCATCCTTTTTCGCCT	RT-qPCR (mouse)
GBP2 forward	CTATCTGCAATTACGCAGCCT	RT-qPCR (human)
GBP2 reverse	TGTTCTGGCTTCTTGGGATGA	RT-qPCR (human)
UBE2L6 forward	TGGACGAGAACGGACAGATTT	RT-qPCR (human)
UBE2L6 reverse	GGCTCCCTGATATTCGGTCTATT	RT-qPCR (human)
STAT1 forward	CAGCTTGACTCAAAATTCCTGGA	RT-qPCR (human)
STAT1 reverse	TGAAGATTACGCTTGCTTTTCCT	RT-qPCR (human)
STAT3α/β forward	TGCAGCAATACCATTGACCT	RT-qPCR (human)
STAT3α reverse	AGATTGCTCAAAGATAGCAGAAGT	RT-qPCR (human)
STAT3β reverse	GTTTATCTGTGTGACACCATTCAT	RT-qPCR (human)
GAPDH forward	TCTCCTCTGACTTCAACAGCG	RT-qPCR (human)
GAPDH reverse	ACCACCCTGTTGCTGTAGCC	RT-qPCR (human)

<u>Supplementary Table 3</u>: List of antibodies used in this study. *Cell Signaling Technology (Danvers, MA, USA). **TonBo Biosciences (Cytek Biosciences, CA, USA).

Target	Name	Company	Article#	Method
STAT3	Stat3 (D3Z2G) Rabbit mAb	Cell Signaling*	12640S	Western blot
STAT3 Tyr705	P-Stat3 (Y705) (D3A7) XP ® Rabbit mAb	Cell Signaling*	9145S	Western blot
STAT3 Ser727	P-Stat3 (S727) (D8C2Z) Rabbit mAb	Cell Signaling*	94994S	Western blot
STAT1	Stat1 Rabbit Ab	Cell Signaling*	9172P	Western blot
ACTIN	Beta-Actin Rabbit Ab	Cell Signaling*	4967S	Western blot
HRP-linked Antibody	Anti-rabbit IgG HRP-linked Antibody	Cell Signaling*	7074S	Western blot
CD16/CD32	TruStain FcX™ anti-mouse CD16/32, clone: 93	BioLegend	101320	Flow cytometry
CD3	Anti-mouse CD3 violetFluor™ 450, clone: 17A2	TonBo**	75-0032-U100	Flow cytometry
Ter-119	Anti-mouse TER-119 violetFluor™ 450, clone: TER-119	TonBo**	75-5921-U10	Flow cytometry
CD19	Anti-mouse CD19 violetFluor™ 450, clone: 1D3	TonBo**	75-0193-U100	Flow cytometry
c-kit (CD117)	PE/Cyanine5 anti-mouse CD117 (c-kit), clone: 2B8	BioLegend	105810	Flow cytometry
Sca-1 (Ly-GA/E)	PE/Cyanine7 anti-mouse Ly-6A/E (Sca-1), clone: D7	BioLegend	108114	Flow cytometry
CD11b	Anti-mo CD11b, eBioscience™ PE, clone:M1/70	eBioscience	12-0112-82	Flow cytometry
Gr-1 (Ly-6G)	Anti-mouse Ly-6G (Gr-1) violetFluor™ 450, clone: RB6-8C5	TonBo**	75-5931-U100	Flow cytometry
IFNAR-1	PE anti-mouse IFNAR-1 Antibody, clone: MAR1-5A3	BioLegend	127312	Flow cytometry
7-AAD	eBioscience™ 7-AAD Viability Staining	eBioscience	00-6993-50	Flow cytometry
Annexin V	Pacific Blue™ Annexin V	BioLegend	640917	Flow cytometry

Edtmayer et al. Supplementary Figure 1

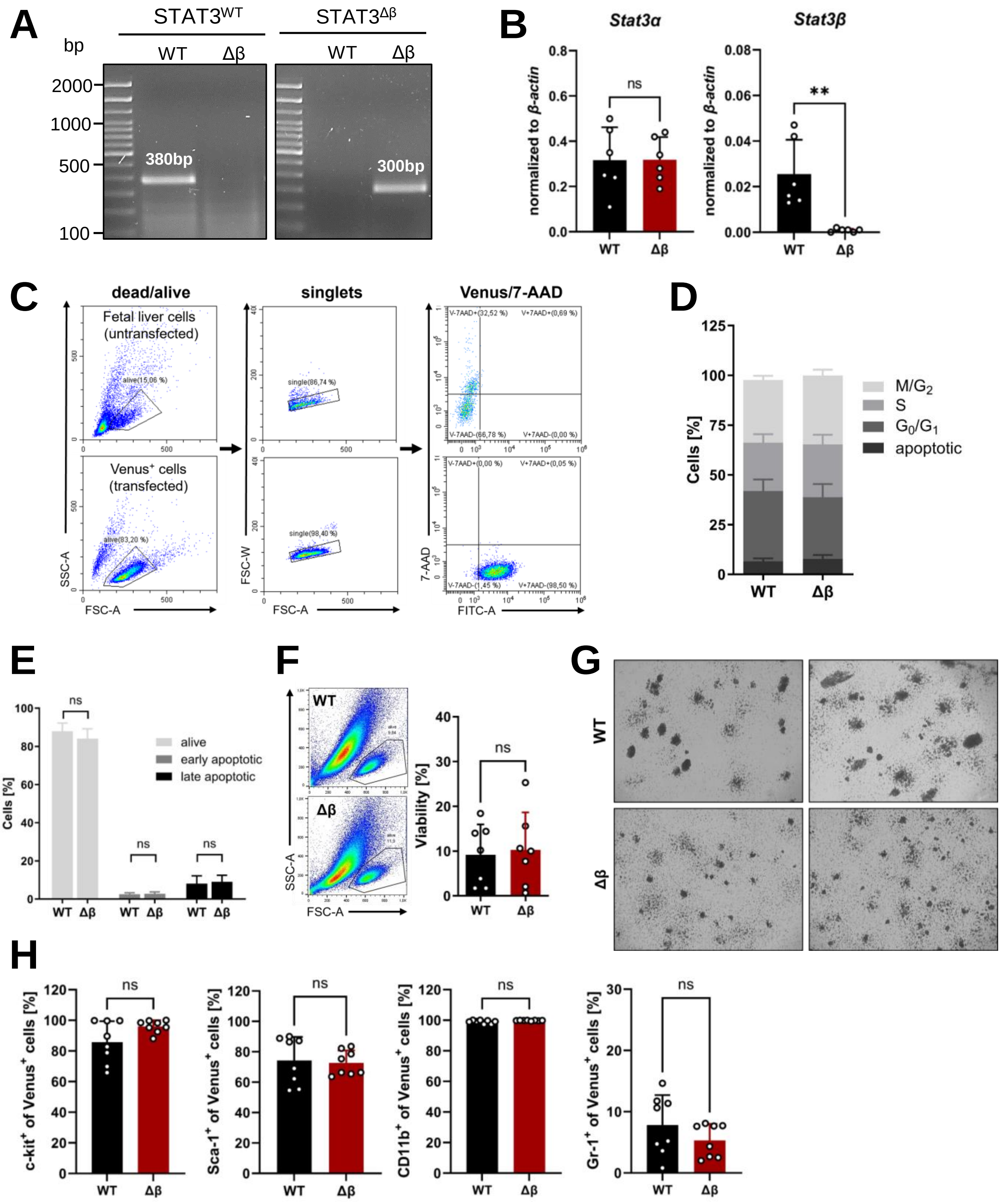

Supplementary Figure 1: STAT3β deficiency does not affect cell cycle and apoptosis *in vitro*. (**A**) Genotyping PCR products of the used animal offspring and cells. (**B**) RT-qPCR analysis of *Stat3α* and *Stat3β* expression in STAT3β-deficient leukemia cells (n=6, 3 independent experiments, 2 different cell lines). (**C**) Gating strategy for 7-AAD$^-$Venus$^+$ cells and untransfected fetal liver cells as negative control analyzed *via* flow cytometry. (**D**) Cell cycle analysis *via* flow cytometry using 7-AAD in suspension culture (n=6, 3 independent experiments, 2 cell lines). (**E**) Apoptosis analysis *via* flow cytometry using Annexin V and 7-AAD in suspension culture (n=8, 3 independent experiments. 2-3 cell lines). (**F**) Viability according to FSC and SSC after 7 days in methylcellulose with representative dot plots (n=6, 2 independent experiments with 3 serial replating). (**G**) Immunophenotyping of *MLL-AF9* transformed FLCs *via* flow cytometry (n=8, 3 independent experiments, 2-3 cell lines). Statistical analysis was performed using Student's *t-test*. p≤ 0.01:**. Error bars represent means ± SD

Edtmayer et al. Supplementary Figure 2

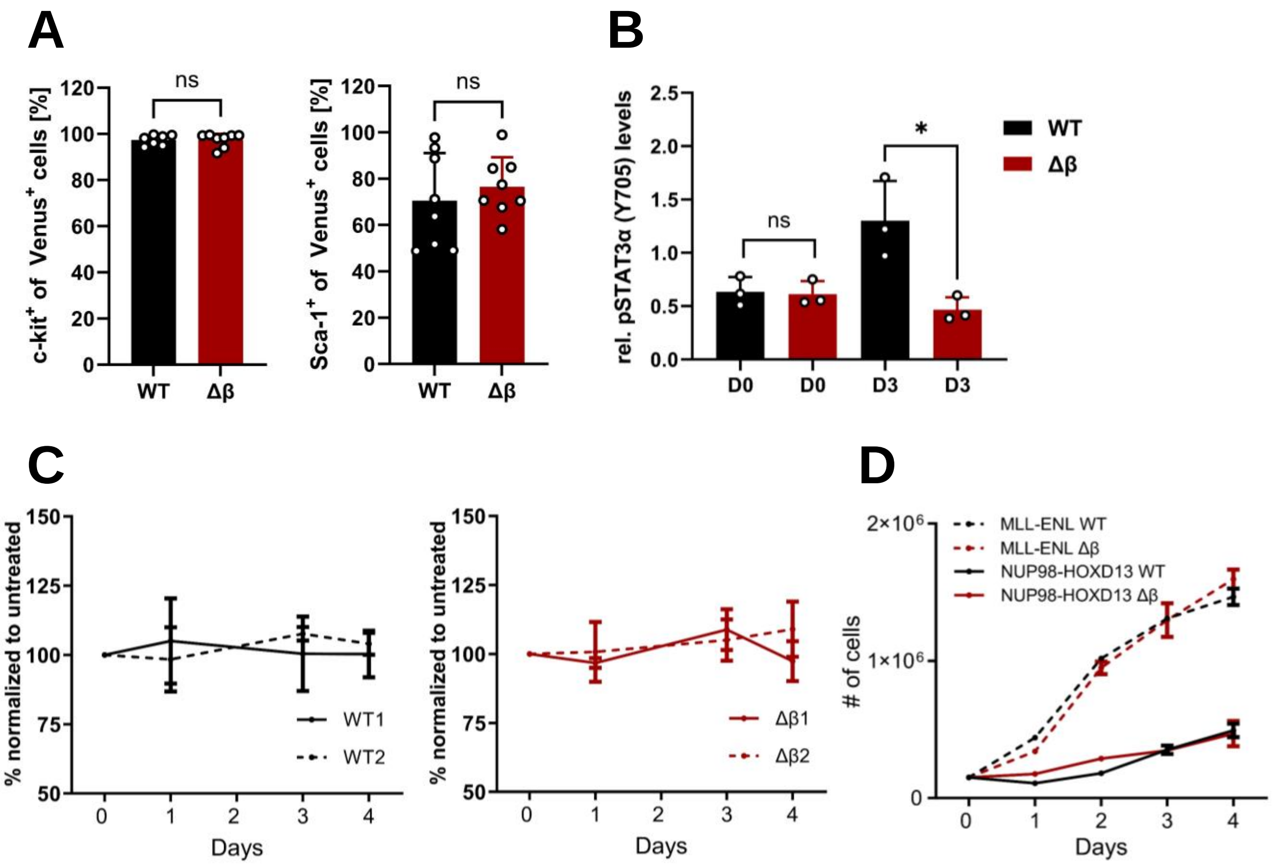

Supplementary Figure 2: G-CSF stimulation does not affect proliferation of leukemia cells. (**A**) Immunophenotyping after 10 days of G-CSF stimulation *via* flow cytometry (n=8, 3 independent experiments, 2-3 cell lines). (**B**) Western blot quantification of Y705 phosphorylation on STAT3α after 3 days of G-CSF stimulation normalized to ACTIN (n=3, independent experiments). (**C**) Representative growth curves of *MLL-AF9* transformed FLCs in the presence of 10ng/ml G-CSF (2 experiments, 3 different cell lines). (**D**) Representative growth curves of *MLL-ENL* and *NUP98-HOXD13* transformed FLCs (n=2, 2 independent experiments). Statistical analysis was performed using Student's *t-test.* p ≤0.05:*. Error bars represent means ± SD.

Edtmayer et al. Supplementary Figure 3

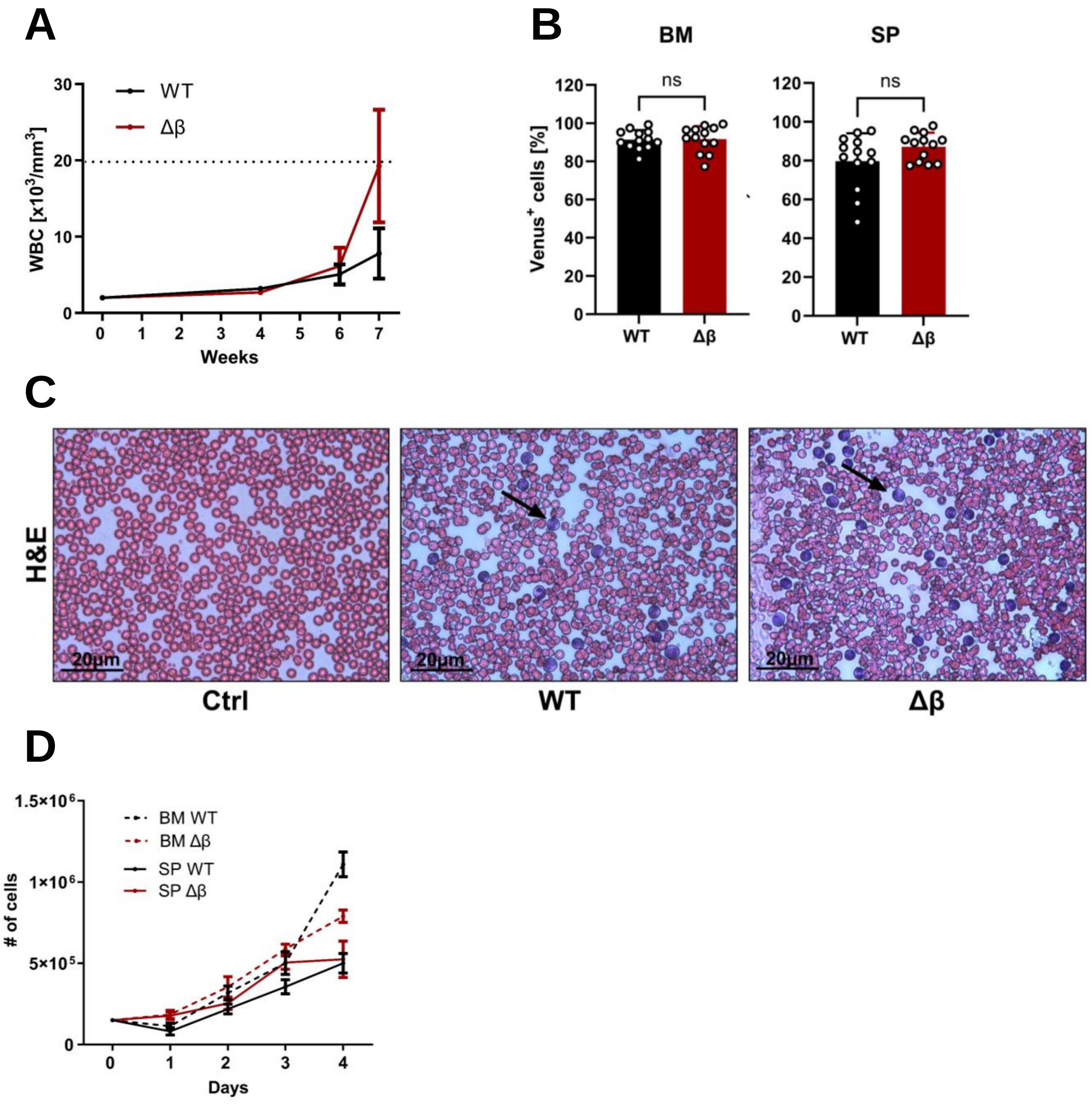

Supplementary Figure 3: Absence of STAT3β accelerates disease progression *in vivo*. (**A**) WBC was monitored for 7 weeks post transplantation (one representative experiment, n≥3 per group). (**B**) Quantification of infiltrating blasts (Venus⁺) in the BM and SP of diseased animals *via* flow cytometry (n=13). (**C**) Representative H&E-stained blood smears of a healthy control (Ctrl) and diseased animals (40x magnification). Black arrows indicate blasts. (**D**) Proliferation of BM- and SP-derived *ex vivo* cells *in vitro* (n=3, 3 different cell lines per group). Error bars represent means ± SD. (H&E Hematoxylin and eosin)

Edtmayer et al. Supplementary Figure 4

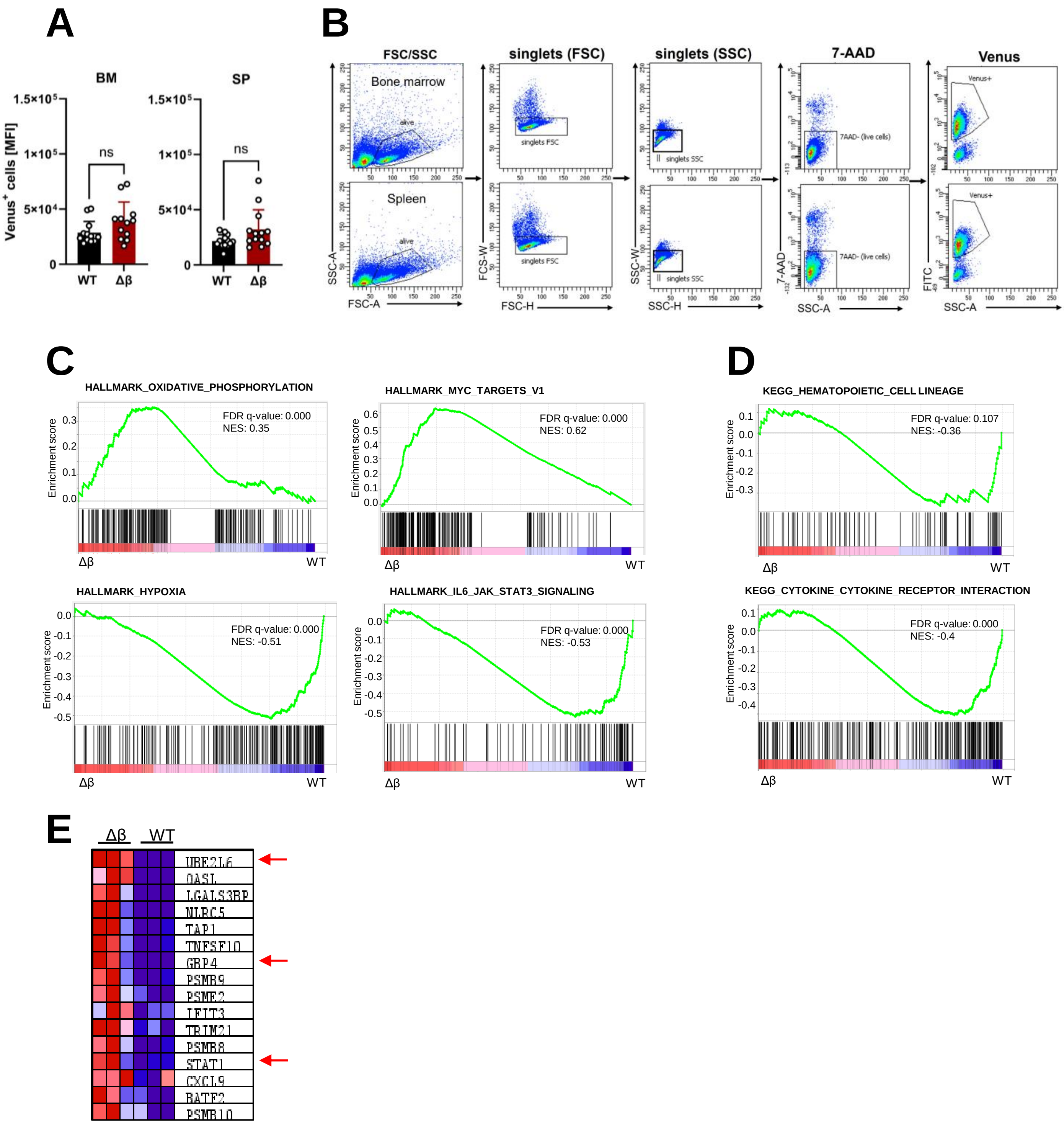

Supplementary Figure 4: Gene expression analysis of blasts isolated from diseased animals. (**A**) MFI of Venus^{+} blasts derived from BM and SP of diseased animals analyzed *via* flow cytometry (n=12). (**B**) Sorting strategy for Venus^{+} cells from BM and SP of diseased animals. (**C**) GSEA of leukemic blasts lacking STAT3β compared to WT cells isolated from SP of diseased animals (FDR False discovery rate, NES normalized enrichment score). (**D**) KEGG pathway analysis of blasts lacking STAT3β compared to WT. (**E**) Heat map of top-ranked genes in the IFN signatures from SP-derived blasts (core enrichment, rank in metric score >1). Statistical analysis was performed using Student's *t-test.* Error bars represent means ± SD. (GSEA Gene set enrichment analysis, KEGG Kyoto Encyclopedia of Genes and Genomes, MFI Mean fluorescence intensity)

Edtmayer et al. Supplementary Figure 5

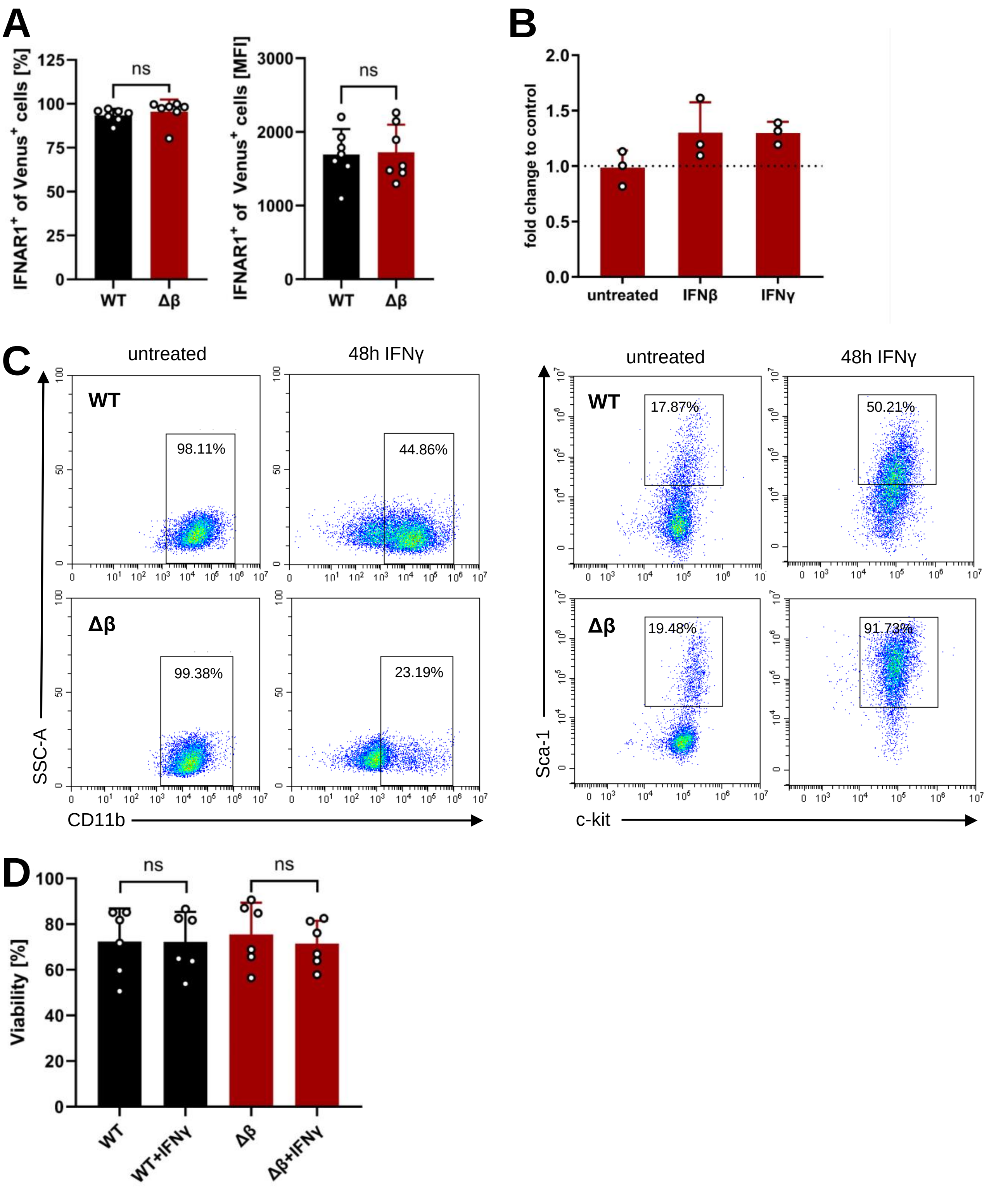

Supplementary Figure 5: STAT3β-deficiency increases IFN responsiveness. (**A**) Expression of the surface receptor IFNAR1 analyzed *via* flow cytometry (n=7, 3 independent experiments, 2-3 different cell lines). (**B**) Western blot quantification of STAT1 protein expression in STAT3β-deficient leukemia cells compared to WT cells after 24h IFNβ or IFNγ stimulation. Quantification of 3 independent experiments. (**C**) Representative dot plots of 48h IFNγ treated leukemia cells. Left: CD11b, Right: LSK. (**D**) Viability (according to FSC and SSC) after 48h IFNγ treatment with representative dot plots (n=6, 3 independent experiments, 2 different cell lines). Statistical analysis was performed using Student's *t-test.* Error bars represent means ± SD.

Edtmayer et al. Supplementary Figure 6

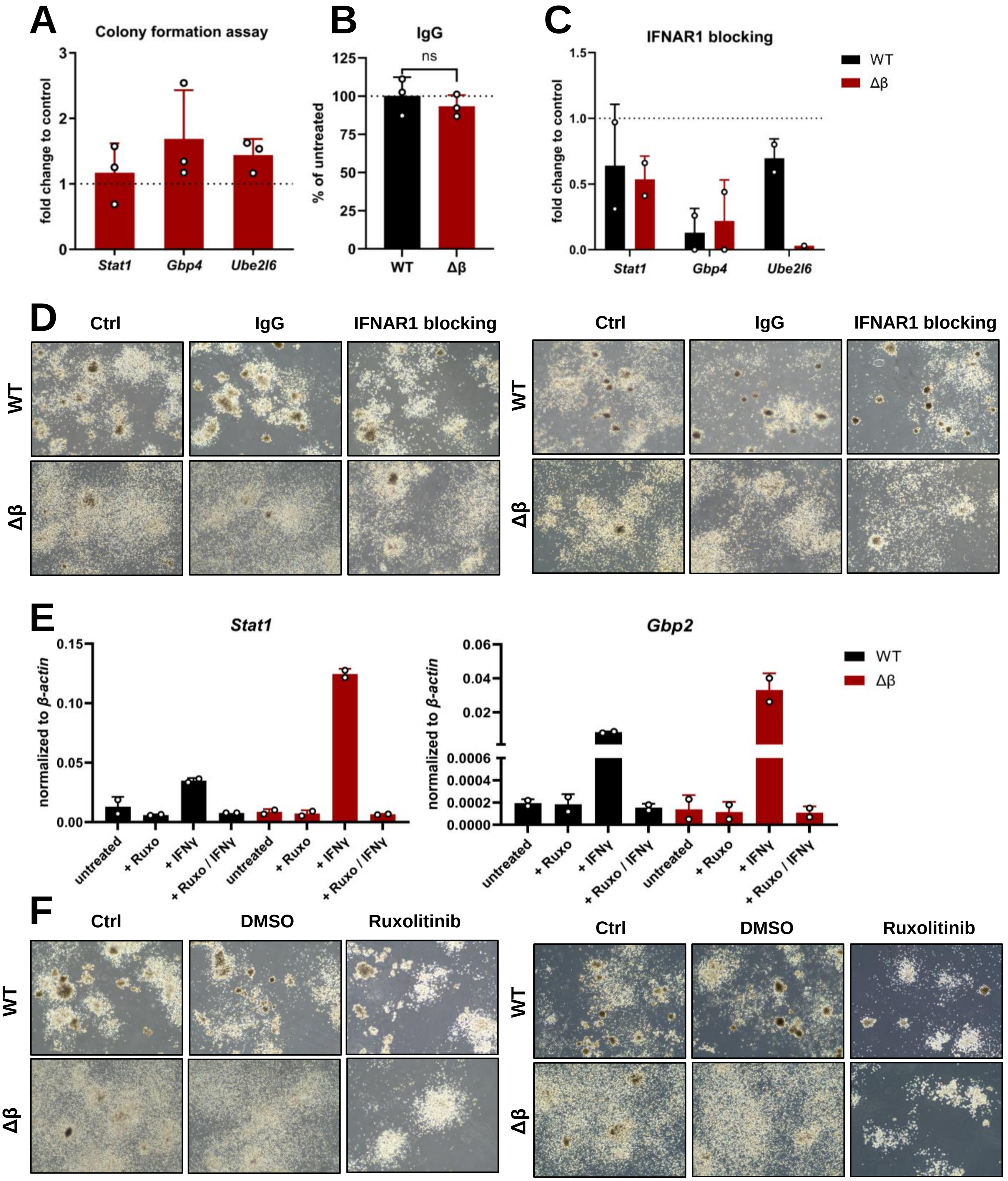

Supplementary Figure 6: Ruxolitinib effectively blocks IFN signaling in leukemia cells. (**A**) RT-qPCR confirms expression of IFN-inducible genes in colonies formed by leukemia cells after 7 days in methylcellulose (n=3, independent experiments). (**B**) CFA in the presence of 2µg/ml isotype (IgG) control normalized to untreated. (n=3, independent experiments). (**C**) Reduced expression of IFN-inducible genes after 7 days in methylcellulose in presence of IFNAR1 blocking analyzed via RT-qPCR (n=2, independent experiments). (**D**) Additional representative pictures of colonies formed in presence of IFNAR1 blocking antibody (2 independent experiments). (**E**) RT-qPCR confirms that IFNγ response is efficiently blocked by Ruxolitinib (Ruxo). Leukemia cells were treated with 5µM Ruxolitinib for 30min followed by 3h IFNγ stimulation (100ng/ml) (n= 2, independent experiments). Data were not statistically compared. Error bars represent means ± SD. (**F**) Additional representative pictures of colonies formed in presence of 5µM Ruxolitinib (2 independent experiments).

Edtmayer et al. Supplementary Figure 7

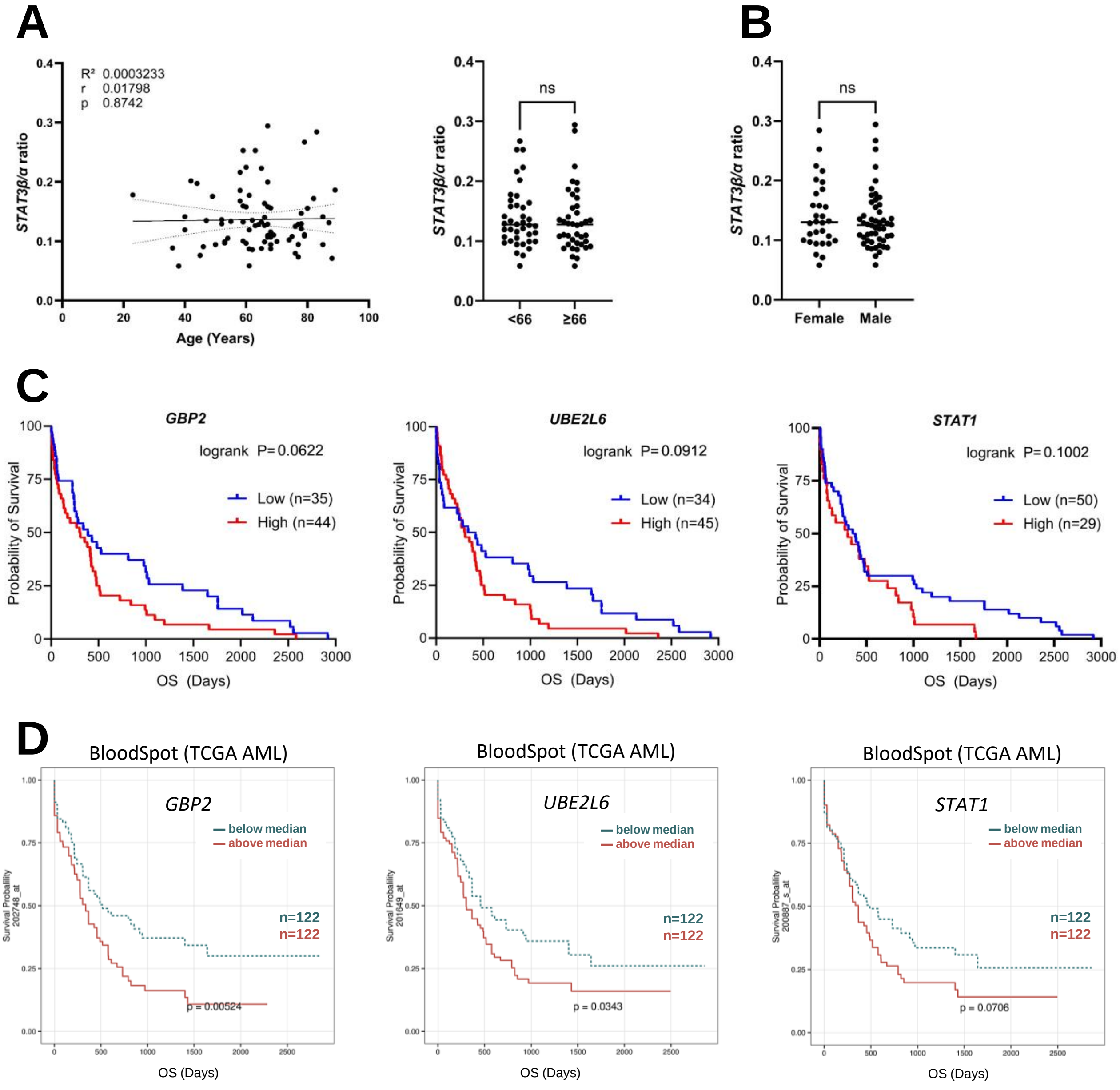

Supplementary Figure 7: Analysis of IFN-inducible genes in AML patients. (**A**) Pearson correlation of *STAT3β/α* mRNA ratio with age of patients at time of diagnosis (n=79). (**B**) *STAT3β/α* mRNA ratio according to the patient gender (females: n=29, males: n=50). Data was analyzed using Student's t-test. (**C**) Expression of IFN-inducible genes (*GBP2, UBE2L6, STAT1*) correlated with survival in AML patient samples (n=79). Gene expression was measured *via* RT-qPCR and normalized to GAPDH. Patients were stratified according to the best cut-off value. Statistical analysis was performed using log-rank (Mantel-Cox) test. (**D**) Kaplan-Meier analysis of IFN-inducible genes using the publicly available AML TCGA dataset (Affymetrix U133 Plus 2) of 244 patients accessed *via* BloodSpot. Patients were stratified based on gene expression above or below median. (TCGA The Cancer Genome Atlas)
