# Supplementary figures and images for: A novel function of STAT3β in suppressing interferon response improves outcome in acute myeloid leukemia
Source: Cell Death Dis. 2024 May 28;15(5):369. doi: 10.1038/s41419-024-06749-9 (PMC11133483; doi:10.1038/s41419-024-06749-9)

Edtmayer et al. - Original Western Blots

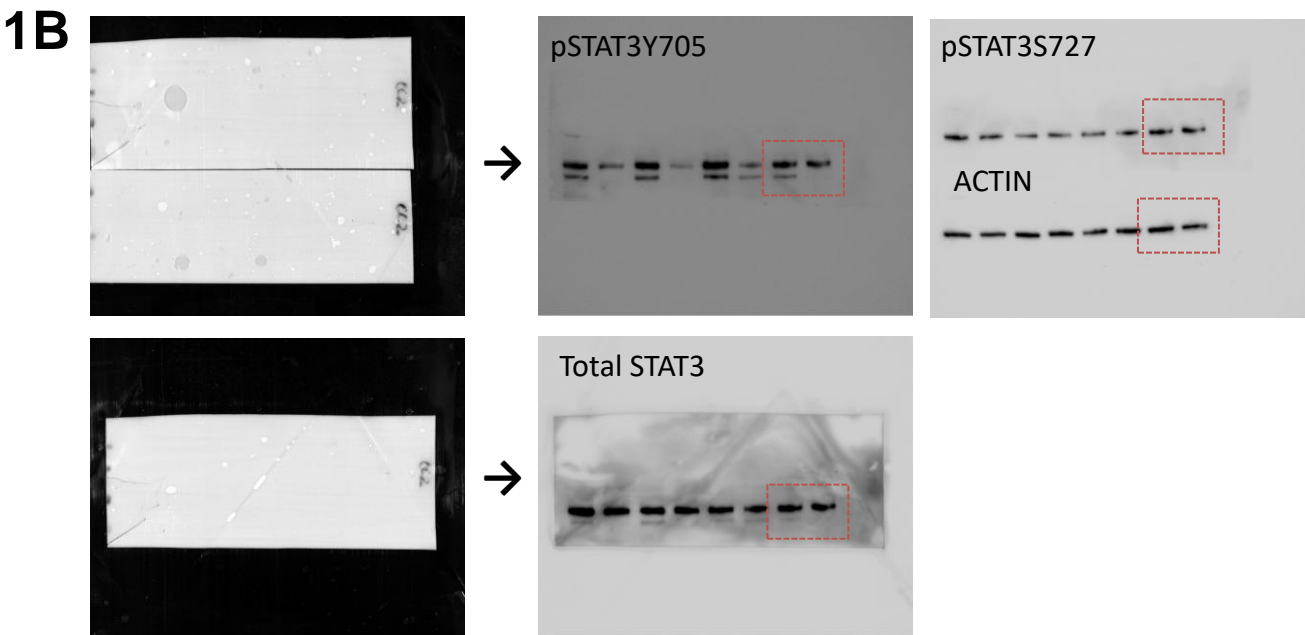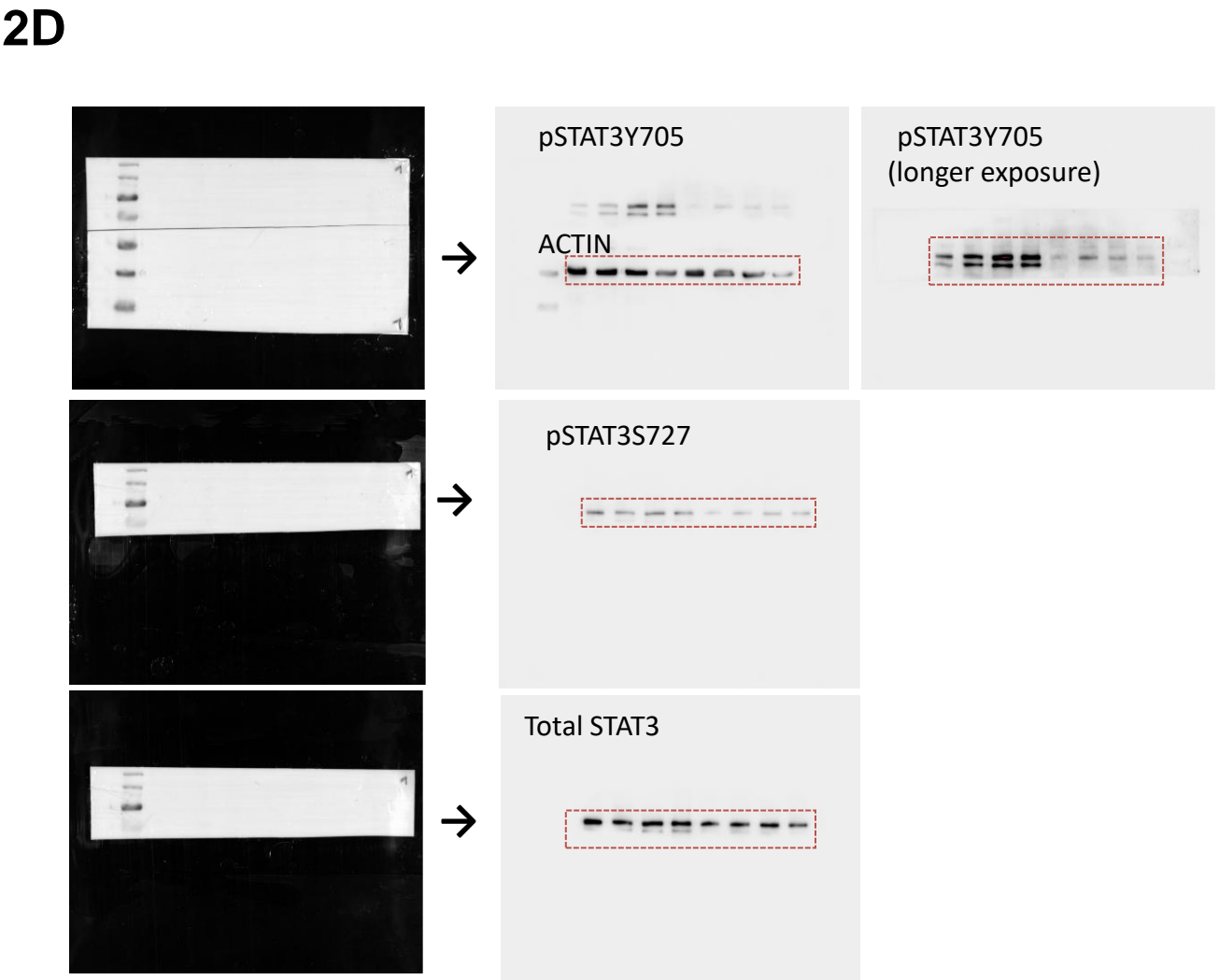

5C

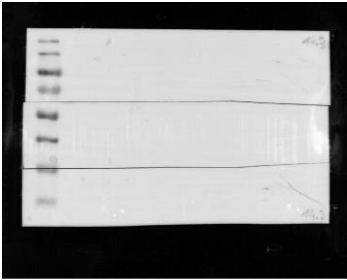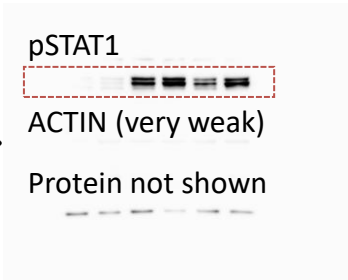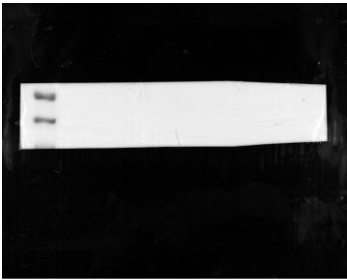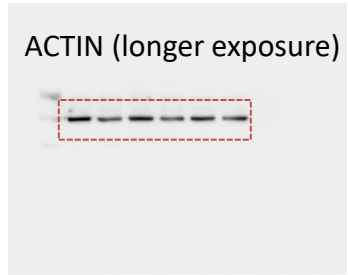

Supplement: Supplementary file 2 — Original Western Blots [file 41419_2024_6749_MOESM2_ESM.pdf]
